# Assessment of climate change effects on vegetation and river hydrology in a semi-arid river basin

**DOI:** 10.1371/journal.pone.0271991

**Published:** 2022-08-29

**Authors:** Jamal Hassan Ougahi, Mark E. J. Cutler, Simon J. Cook

**Affiliations:** Energy, Environment and Society, School of Humanities, Social Sciences and Law, University of Dundee, Dundee, United Kingdom; University of Kashmir, INDIA

## Abstract

Climate change plays a key role in changing vegetation productivity dynamics, which ultimately affect the hydrological cycle of a watershed through evapotranspiration (ET). Trends and correlation analysis were conducted to investigate vegetation responses across the whole Upper Jhelum River Basin (UJRB) in the northeast of Pakistan using the normalized difference vegetation index (NDVI), climate variables, and river flow data at inter-annual/monthly scales between 1982 and 2015. The spatial variability in trends calculated with the Mann-Kendall (MK) trend test on NDVI and climate data was assessed considering five dominant land use/cover types. The inter-annual NDVI in four out of five vegetation types showed a consistent increase over the 34-year study period; the exception was for herbaceous vegetation (HV), which increased until the end of the 1990s and then decreased slightly in subsequent years. In spring, significant (p<0.05) increasing trends were found in the NDVI of all vegetation types. Minimum temperature (Tmin) showed a significant increase during spring, while maximum temperature (Tmax) decreased significantly during summer. Average annual increase in Tmin (1.54°*C*) was much higher than Tmax (0.37°*C*) over 34 years in the UJRB. Hence, Tmin appears to have an enhancing effect on vegetation productivity over the UJRB. A significant increase in NDVI, Tmin and Tmax during spring may have contributed to reductions in spring river flow by enhancing evapotranspiration observed in the watershed of UJRB. These findings provide valuable information to improve our knowledge and understanding about the interlinkages between vegetation, climate and river flow at a watershed scale.

## Introduction

The Hindu Kush Himalaya (HKH) region is an area of rapid change due to climate change and human-induced land cover modifications [1]. The structure and functioning of terrestrial ecosystems are driven largely by changing dynamics of vegetation, which in turn are greatly affected by global environmental changes [2]. In northern areas of Pakistan, research on vegetation dynamics is limited due to the lack of field-based biophysical observations. Remotely sensed data can provide invaluable information on the spatiotemporal patterns of, and linkages between, climate, hydrology, and vegetation cover.

The HKH region is the main source of freshwater of South Asia and parts of southeast Asia [3]. The ten largest river systems in Asia are fed by water originating from snow, glaciers and rainfall [4], and these rivers in turn are crucial for ecosystem functioning, water supply, and agriculture [5]. The hydrological cycle is becoming more unpredictable, resulting in devastating impacts, such as landslides, droughts and catastrophic floods [6]. Studies of long-term climate change, hydrology, and vegetation cover at the basin scale are therefore valuable in understanding the challenges and threats to areas affected by changes in the hydrological cycle.

The influence of water project construction (dams and floodgates) on river flow, ecology and surrounding environment is crucial in river basin management. These schemes could have an impact on quantity and quality of the river flow. According to [7], dams and floodgates reduced the average annual river flow by 2% in the Huai River Basin. However, the influence of artificial factors such as existed reservoirs and floodgates upstream of the UJRB are excluded. Most of the dams in the UJRB (i.e., Azad Pattan Hydropower project, Karot Hydropower project and Kohala Hydropower project) are scheduled for completion after 2022. Only Uri dam was built in 2014 which is a run-of-the-river type project with 10 km tunnel. This study is based on the data during 1982 to 2015 which exclude the influence of any artificial factors such as water conservancy projects on the river flow of the UJRB.

Mountain ecosystems are some of the most sensitive to climate change [8], yet the effect of environmental change to these ecosystems remains poorly understood [9]. There has been a great deal of research in the HKH region that has focused on understanding the impact of climate change on glaciers [10–13], changes in glacier-related water resources [14–16] hydrological risks [17, 18] or monsoon-driven run-off dynamics [19, 20]. Despite the coupling of ecology and hydrology, ecological changes at high altitude in the HKH region have been somewhat overlooked [21]. A first step towards understanding the role of vegetation in ecohydrology of the HKH would be to investigate vegetation cover trends [10].

Climate variability is one of the main drivers of vegetation dynamics [22, 23], affecting the development, growth and spatial distribution of vegetation [24, 25]. In turn, vegetation cover exerts control on climate by alteration of the biophysical characteristics of the land surface such as albedo and roughness [26]. Some climate models showed a reduction in temperature-limited ecosystems in the HKH region over the next 50–100 years, thus generating extra space for vegetation expansion in the future [9]. Ecosystem changes are associated with strong climate warming, as evidenced in the northern regions of the Earth, such as earlier and longer growing seasons, northward shift in vegetation biomes [27], tundra shrub expansion [28], increases in productivity [29, 30] and greening of landscape [31]. However, some recent studies have showed vegetation greening globally, potentially due to elevated CO2 fertilization [32], which may indicate changing productivity and impact on eco-hydrological responses. However, whether such changes are long-term or short-term responses remains unclear. [33] suggested that global annual photosynthesis has increased by 11.85±1.4% between 1981 to 2020.

Enhanced evapotranspiration rates and dissipation of more than half of solar energy absorbed by the Earth have also contributed to local cooling in different parts of the world [34]. At zero terrestrial evapotranspiration, the Northern Hemisphere would be 15–25°C warmer, meaning that evapotranspiration associated with vegetation growth results in cooling local temperatures [35]. The soil-vegetation-atmosphere system relates to a key ecological process of evapotranspiration and any changes in evapotranspiration will strongly affect the distribution of water resources in a watershed, especially in arid and semiarid regions [36]. ET is controlled by available soil moisture and topography [37], climate and vegetation characteristics [38]. Trends in temperature during the summer showed a significant cooling in the Upper Jhelum River Basin (UJRB) during 1961 to 2013 [39]. In this context, vegetation feedbacks and their ability to impact local temperatures and river flow is important in interpreting climate warming projections and adopting measures to safeguard ecosystems.

Greening and browning of vegetation are commonly used to assess the productivity of natural and agricultural lands [40–43]. Vegetation indices are an efficient way of estimating photosynthesis dynamics by using red and infrared portions of the electromagnetic spectrum [44]. The NDVI is a measure of greenness which has been strongly correlated with vegetation productivity [40, 41] as well as to estimate a range of biophysical variables, such as land cover characteristics, leaf area and canopy coverage, as well as vegetation dynamics [45–48].

The responses of vegetation at regional scales can be monitored with the application of remote sensing. In many investigations, the relationship between NDVI and climate has been discussed, covering different geographic areas and ecosystems, but is still far from being fully understood [49, 50]. Some researchers have studied the relationship between climate factors and NDVI-based vegetation changes in river basins of the Himalayas. For example, [51] studied the spatiotemporal NDVI based vegetation changes and their response to climate in the Koshi River Basin of middle Himalaya. They found that the NDVI significantly increased in 1982–1994, 2000–2006, 2000- 2011 and decreased during 1994–2000. They suggested that temperature and precipitation were the major driver of change. [52] studied the NDVI of forests and grassland in the upper catchment of the Yellow river. A strong correlation was found between NDVI values and climatic indices. [53] characterized vegetation changes on the Tibetan Plateau and found a positive correlation between NDVI and temperature, while the relationship between precipitation and NDVI was complex. Thus the NDVI provides a reasonable measure of vegetation dynamics, which has been linked to climatic variables in the region.

Vegetation also has an important role in influencing the monsoon [54]. The energy exchange between vegetation and atmosphere affects air mass movement and can lead to warming or cooling at the surface of the Earth [55]. The climatic trends revealed asymmetric warming over the UIB where Tmin increases significantly throughout the year, while Tmax shows a significant decrease during the summer [56]. In this context, vegetation responses to asymmetric warming trends along with river flow variations are important to understand energy fluxes. This study aims to investigate whether climate variables and river flow regime are potentially influenced by vegetation growth and vice versa at the catchment scale. The specific objectives are:

1. To analyse the long-term trends and spatiotemporal variations in NDVI, precipitation and temperature over UJRB.
2. To evaluate the effect of climate variability on vegetation growth at annual and seasonal scales; and
3. To analyse whether vegetation growth shows any relationship with river flow over the study period (1982–2015).

## Materials and methods

### Study area

**Fig 1:**
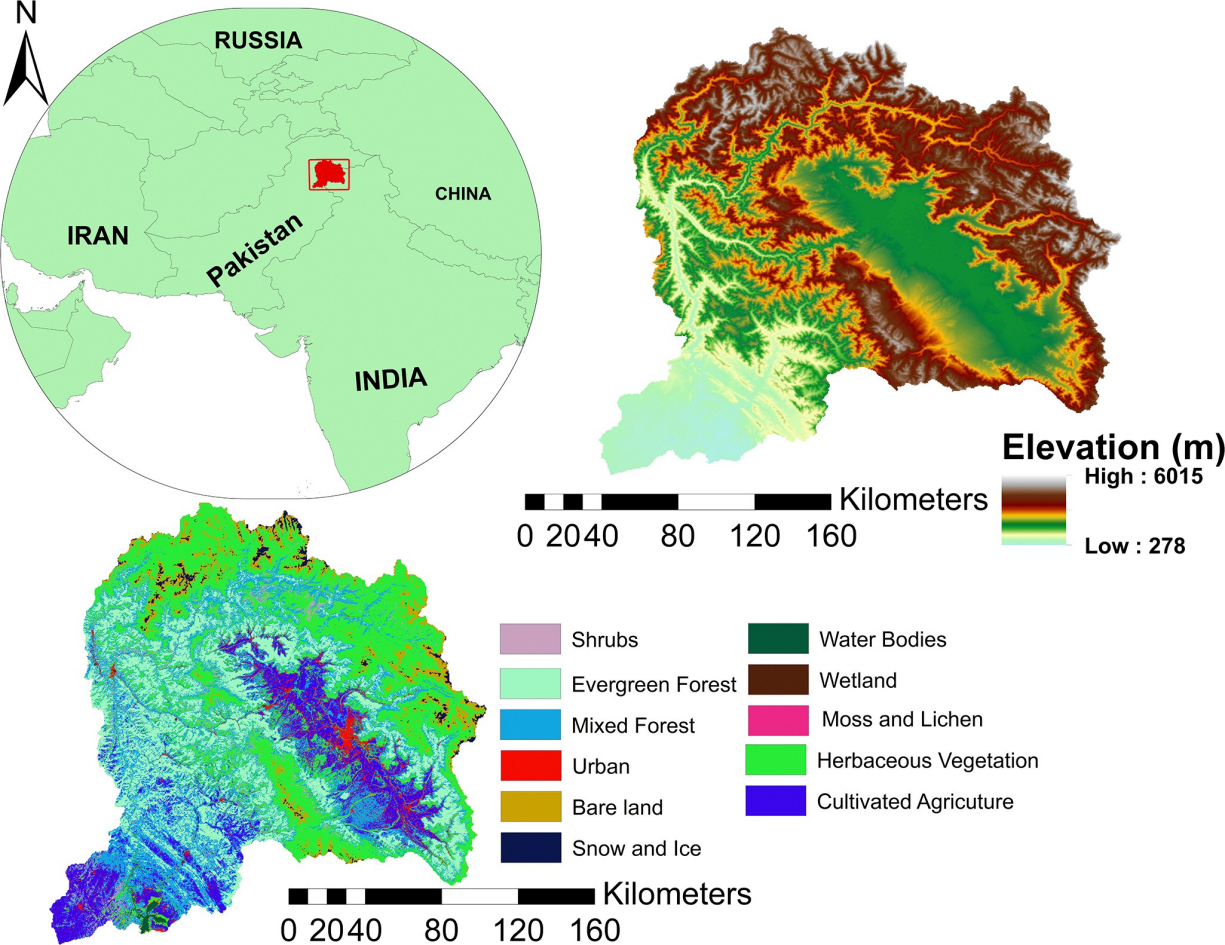
The location, elevation and land use/cover of the study area.

The Upper Jhelum River Basin (UJRB) is a part of the Upper Indus River Basin located between 33°4’-35°10’N latitude to 73°8’-75°35’E longitude (Fig 1). The UJRB is an important trans-boundary basin between Pakistan and India covering a large area in the western Himalayas. The total area of the UJRB is 48,718Km^2^. The Verinag spring, in the north-west side of Pir Panjal, is the origin of Jhelum River, which flows parallel to the Indus River at an average elevation of 1700 m. Glaciers are found on the northern side of Kashmir valley and also contribute water to the Jhelum River. There is a steep elevation gradient from north to south which results in great variations in temperature and precipitation.

### Datasets

#### GIMMS NDVI3g data

In this study, the latest version of the Global Inventory Monitoring and Modelling system Normalized Difference Vegetation Index (GIMMS NDVI3g) derived from the Advanced Very High-Resolution Radiometer (AVHRR) satellite sensor was used. The dataset has a length of 34 years covering January 1982 to December 2015, making it appropriate for long-term studies of trends in vegetation, seasonality and coupling between vegetation and climate variability [57]. The spatial and temporal resolution of GIMMS3g is 1/12 degrees and 15 days, respectively. The data are subjected to atmospheric correction, cloud screening, radiometric calibration and solar zenith angle correction to remove effects not associated with vegetation [58].

#### Land use/cover (LULC) and hydrometeorology data

Several global LULC products have been developed from remotely sensed data since the 1980s [59–61]. These products have significantly advanced our knowledge of the Earth [62]; however their application has limitations due to inconsistency among their categorical format [63]. In this study, LULC classes of the UJRB were derived from the Copernicus Global Land Service (CGLS) product (Fig 1). The CGLS provided its first land cover product in July 2017, which was later improved to map the entire globe. The land cover map was derived from the vegetation instrument on board the PROBA satellite at 100 m spatial resolution [64].

TerraClimate is a high resolution (4km) global dataset derived from datasets such as climatological norms of WorldClim, time varying data from CRUTs 4.0 and the Japanese 55-year Reanalysis (JRA55) at monthly time steps. TerraClimate data have been validated from ground station data of annual temperature, precipitation and reference evapotranspiration, as well as annual river flow data from streamflow gauges [65]. The discharge data at Mangla outlet were obtained from the Water and Power Development Authority (WAPDA) in Pakistan.

### Methodology

Datasets were prepared into monthly and quarterly raster time series before applying the Mann-Kendall (MK) trend test. MK trend analysis was used to estimate annual and seasonal trends in NDVI, precipitation, Tmin, and Tmax on area-weighted average climate data over the UJRB from 1982 to 2015. The magnitude of trend was shown by the slope, while the significance of the trend was shown by p-values. Pixel-wise trends were computed from monthly and annual satellite time-series data of NDVI, gridded and reanalysis data of precipitation and temperature executed in R (package: Kendall v2.2, R version:3.4.3).

Monthly Maximum Value Composites (MVCs) were generated from bimonthly data to remove partial effects of clouds, atmosphere and solar altitude. The NDVI values range from -1 to 1; values close to -1 correspond to water, and values close to zero correspond to barren areas. Hence, NDVI values below 0.1 were replaced with “NA” and excluded from the analysis in order to use only NDVI values that represent vegetation response.

Firstly, annual and monthly data were spatially averaged over the UJRB to compute trend statistics. Secondly, trends were computed for each pixel in the satellite NDVI data and gridded precipitation and temperature data. Pixel-wise correlations between NDVI and climatic variables were calculated from aggregated annual and seasonal time-series data over a 34-year period, similar to the analysis proposed by [53, 66, 67]. NDVI values were spatially averaged over the UJRB and all vegetation types were used to extract NDVI values of the specific LULC classes.

A pixel-wise trend estimation and Spearman correlation was applied to the output from monthly time-series data of NDVI and TerraClimate, consisting of 408 layers (grids). First, the NDVI dataset of 8-km spatial resolution was resampled to match the 4-km spatial resolution of the climate dataset. Subsequently, a pixelwise correlation was conducted between the time-series NDVI data and gridded precipitation, Tmin and Tmax data over the UJRB.

The annual and monthly time series of NDVI over the UJRB and vegetation classes such as evergreen forest (EF), cultivated agriculture (CA), shrubs (SH), herbaceous vegetation (HV) and mixed trees (MT) from LULC data were extracted to analyse the relationship between climate features and NDVI. Monthly time-series NDVI data were extracted from MVCs. In order to better understand seasonal contributions, each year was further divided into four quarters considered from January to March as first quarter (Q1) or winter; April to June as second quarter (Q2) or spring; July to September as third quarter (Q3) or summer; and October to December as fourth quarter (Q4) or autumn.

## Results

In this section, results are presented for two key analyses: (1) annual and seasonal trends within spatially averaged data over the whole UJRB region, and (2) pixel-wise analysis of annual and seasonal trends over the UJRB. Similarly, correlation among NDVI and hydrometeorological variables is also presented at a regional scale and on a pixel-wise basis, both at annual and seasonal timescales.

### Regional scale annual and seasonal trends

The modified MK trend test was applied on spatially averaged data on an annual basis over the whole UJRB. Significant trends in annual NDVI for the whole UJRB and vegetation classes ‘cultivated agriculture’ (CA), ‘evergreen forest’ (EF), ‘mixed trees’ (MT), ‘shrubs’ (SH) and ‘herbaceous vegetation’ (HV), and Tmin and river flow were observed during 1982 to 2015 (Table 1). Tmin increased significantly (p<0.05) over the UJRB while non-significant trends were detected in Tmax and precipitation. However, annual river flow decreased significantly during this period. The magnitude of increase in Tmin (1.54°C) was much higher compared to Tmax (0.37°C) over the UJRB during 1982 to 2015.

**Table 1:** Annual Mann-Kendall trend statistics calculated from spatially averaged data of NDVI, precipitation, maximum temperature (Tmax) and minimum temperature (Tmin) for each vegetation type during 1982 to 2015.

Variables	p-value	z-value	Sen’s Slope
Tmax (°C)	0.08	1.7	0.005
Tmin (°C)	<0.05	9.6	0.45
Precipitation (mm)	0.5	-0.7	-0.09
River Flow (*m*^3^/*s*)	<0.05	-3.1	-5.2
NDVI of UJRB	<0.05	13	0.0014
NDVI of CA	<0.05	14	0.0019
NDVI of EF	<0.05	13	0.0015
NDVI of MT	<0.05	12.3	0.002
NDVI of HV	0.2	1	0.0003
NDVI of Shrubs	<0.05	7	0.0007

**Fig 2:**
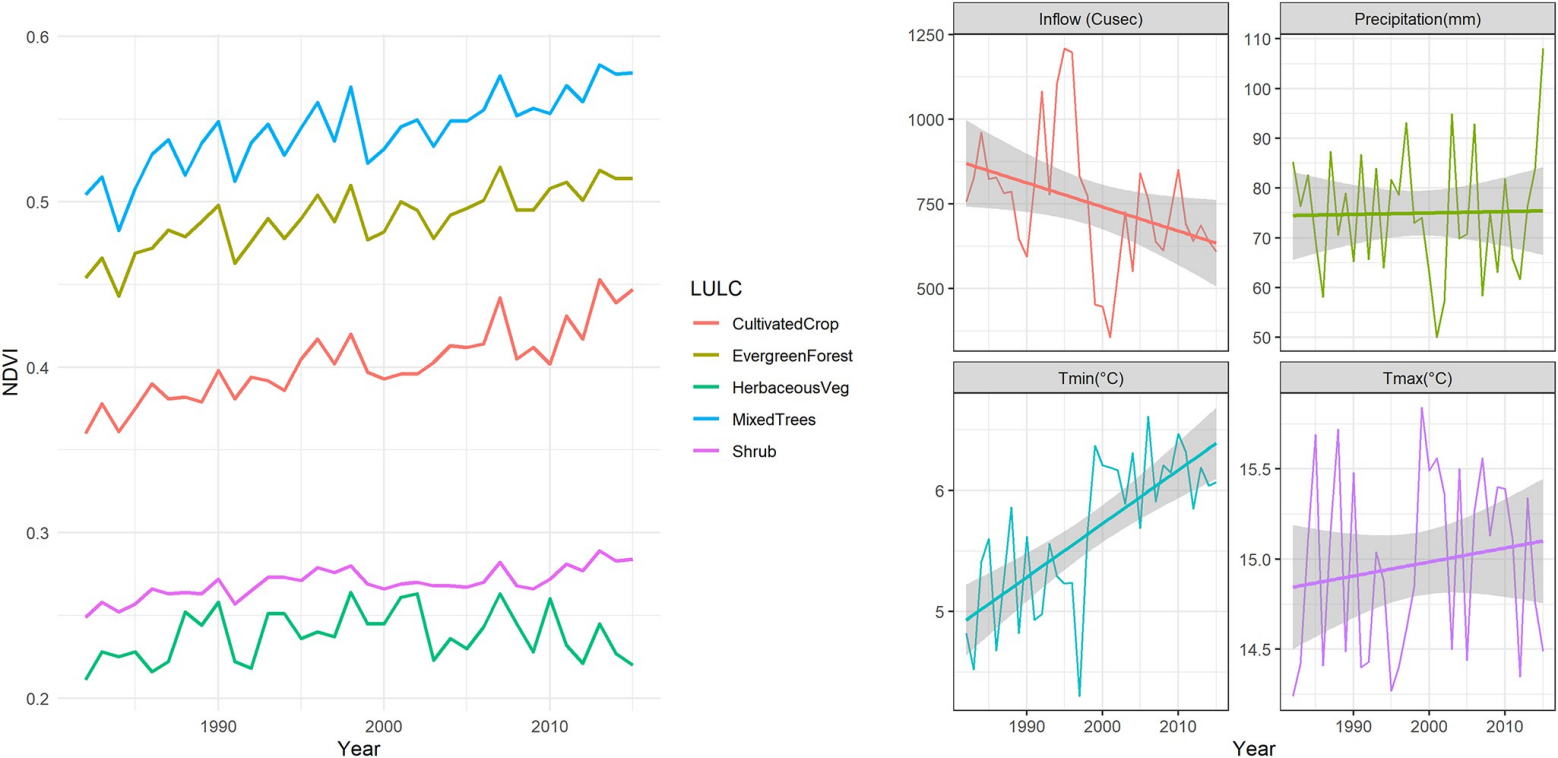
Average annual NDVI and hydrometeorological data variability during 1982–2015.

The inter-annual variability of NDVI and hydrometeorological variables show greater variations over the 34-year study period (Fig 2). Tmin and Tmax show an unusually sharp increase between 1995 to 2001. However, river flow and precipitation showed sharp decreases during this period (1995 to 2001). The NDVI for all vegetation classes showed a consistent increase over 34 years, except for the class HV which showed a significant increase from 1982 to the end of 1990s and then a decrease from 2000 to 2015. Inter-annual variability of NDVI was closely related to temperature with the impact of precipitation seemingly less important (Fig 2).

Seasonal trend statistics of hydro-meteorological variables during 1982 to 2015 showed significant (p<0.05) increases in Tmin during all four quarters of the year (Table 4). However, Tmax decreased significantly in the third quarter (Q3) and increased significantly in the fourth quarter (Q4). Significant decreasing trends in river flow were only detected in the second quarter (Q2), while a non-significant increase was detected during Q1, Q3 and Q4.

The seasonal trend statistics of climatic variables showed non-significant increases in precipitation all year round, while Tmax decreased during the summer and increased during the autumn (Table 4). Seasonal trends in Tmin showed an increase during all four seasons. The highest increase in Tmin was recorded during spring (1.94°C). The NDVI of all vegetation classes (i.e., CA, EF, MT and SH) increased during each quarter except the NDVI of HV, which was non-significant (Table 2).

**Table 2:** Seasonal Mann-Kendall trend statistics calculated from spatially averaged data over the whole UJRB and each vegetation class in four quarters (Q1, Q2, Q3 and Q4) during 1982 to 2013.

Variables	Quarters	p-value	z-value	Sen’s Slope
NDVI of UJRB	Q1	<0.05	12.4	0.0014
	Q2	<0.05	13.7	0.0027
	Q3	<0.05	5.5	0.0011
	Q4	<0.05	5.2	0.0007
NDVI of CA	Q1	<0.05	10	0.0022
	Q2	<0.05	10.3	0.0022
	Q3	<0.05	8	0.0015
	Q4	<0.05	8.1	0.0018
NDVI of EF	Q1	<0.05	5.7	0.0009
	Q2	<0.05	13.6	0.0021
	Q3	<0.05	8.7	0.0014
	Q4	<0.05	9.2	0.0015
NDVI of MT	Q1	<0.05	7.9	0.0017
	Q2	<0.05	9.7	0.0021
	Q3	<0.05	11.2	0.0016
	Q4	<0.05	9.6	0.0018
NDVI of SH	Q1	<0.05	5.6	0.0007
	Q2	<0.05	7.8	0.0009
	Q3	<0.05	6.8	0.0008
	Q4	<0.05	4	0.0003
NDVI of HV	Q1	0.07	-2.7	-0.0003
	Q2	<0.05	3	0.0014
	Q3	<0.05	3.2	0.0008
	Q4	<0.05	-2.9	-0.0009

### Pixel-wise annual and seasonal trends

**Fig 3:**
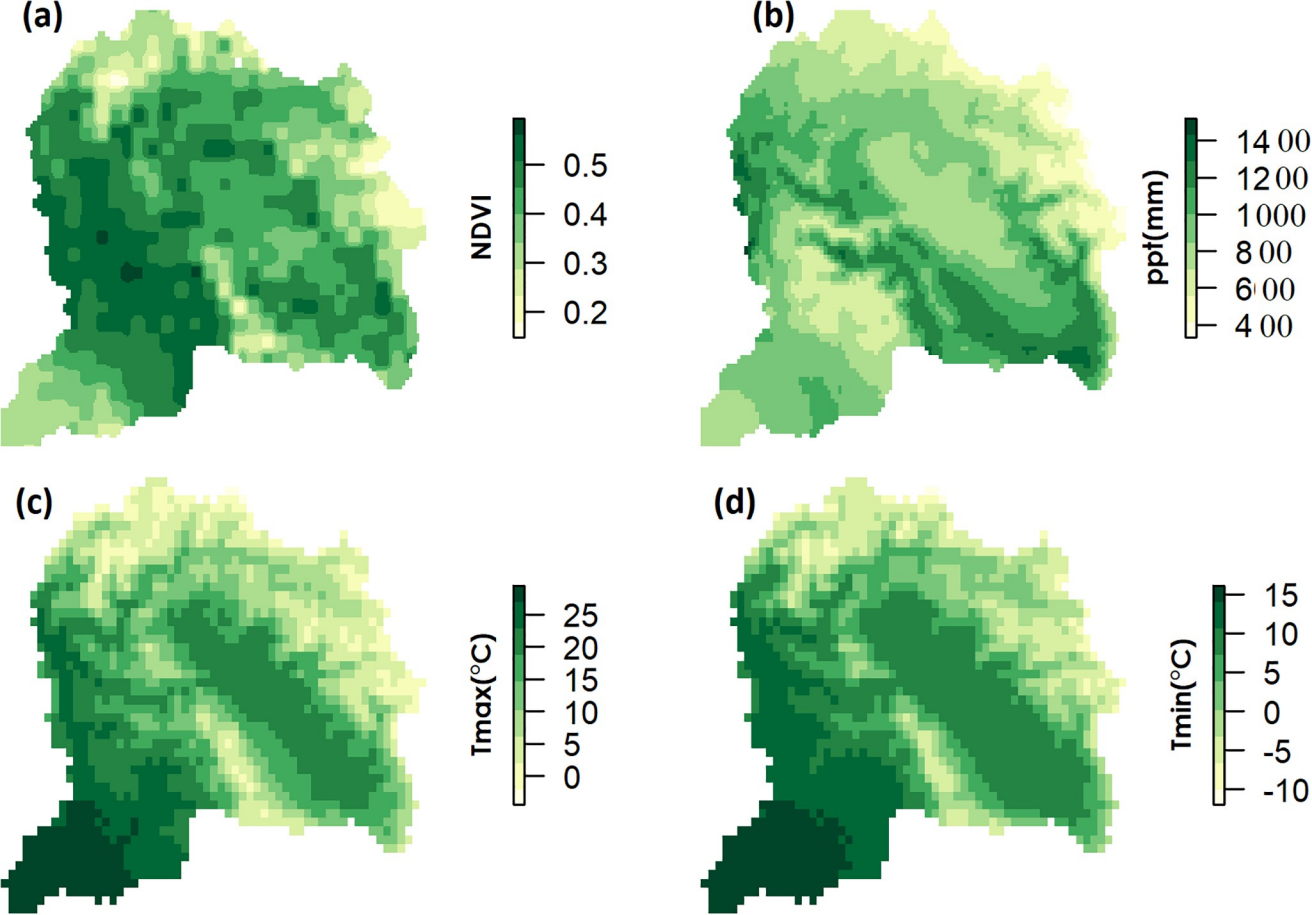
Spatial distribution of average annual NDVI (a), precipitation (b), Tmin (c) and Tmax (d) over the UJRB during 1982 to 2015.

The patterns in spatial distribution of the mean annual NDVI and climatic variables over the UJRB are shown in (Fig 3). NDVI appears more closely related to temperature variations over the whole UJRB. However, NDVI is positively correlated with precipitation in the southern region of the UJRB.

**Fig 4:**
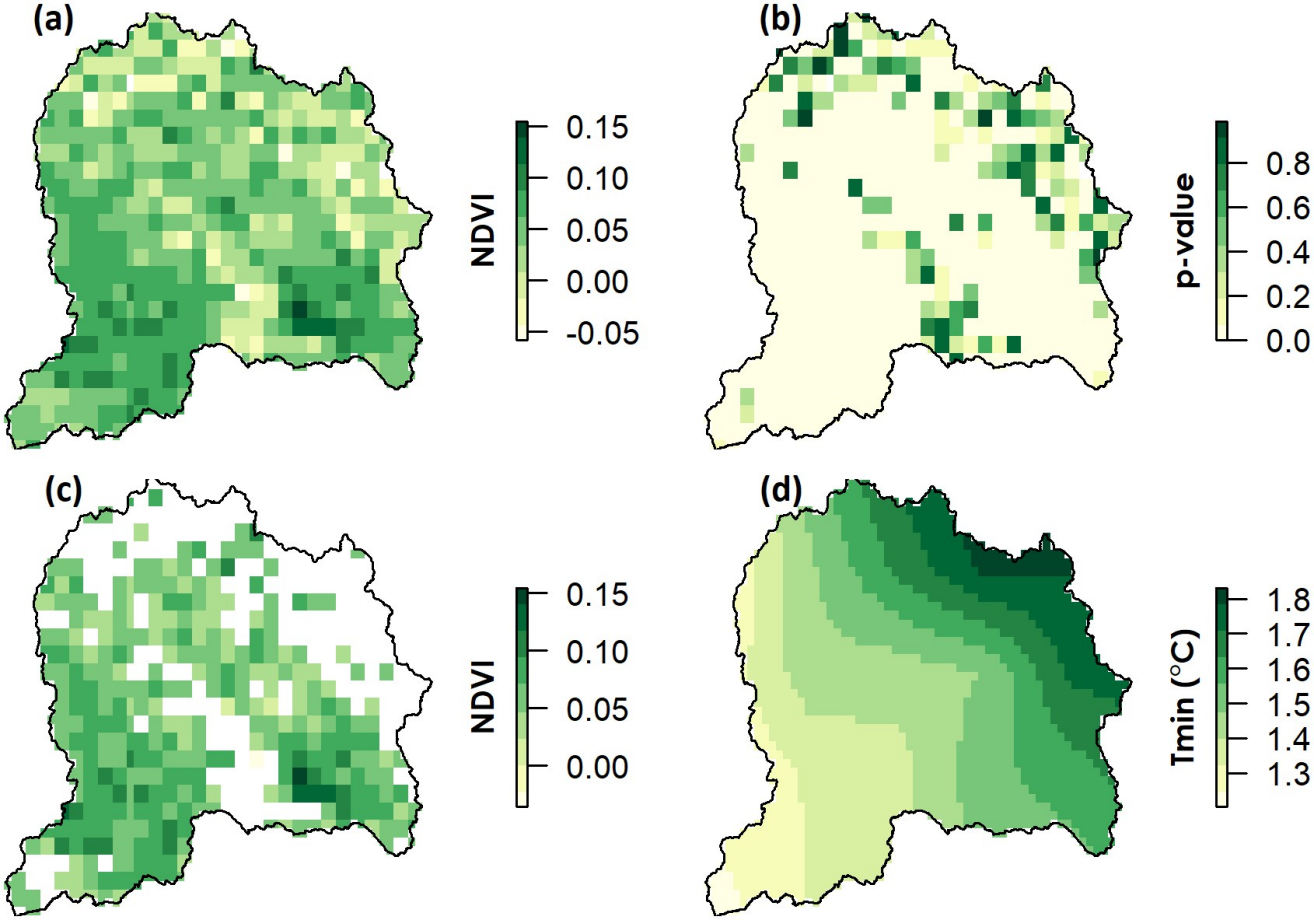
Pixel-wise trends in annual time series data of NDVI and Tmin during 1982 to 2015 (a) Sen’s slope of NDVI (b) p-values of NDVI, (c)significant trends (p<0.05) in average annual slope (p<0.01) of NDVI and (d) significant trends in annual average slope (p<0.05) of Tmin.

In order to assess and quantify annual spatiotemporal patterns and trends, the Sen’s slope between NDVI and the climate variables was computed; only significant trends (p<0.05) in annual slope of NDVI are shown in Fig 4. The spatial pattern of trends, represented by Sen’s slope, were heterogeneous. The spatial variability of annual NDVI trends showed statistically significant (p<0.05) positive trends over most of the UJRB but this was not uniform across the whole catchment (Fig 4).

The influence of climate drivers on vegetation growth varied in different regions of the UJRB. A non-significant change in Tmax and precipitation trends was detected over the UJRB, whereas a significant increase in Tmin was detected (Fig 4). The southern region of the UJRB showed a lower increase in Tmin compared to the northern region where it increased by up to 1.8°C over 34 years.

**Fig 5:**
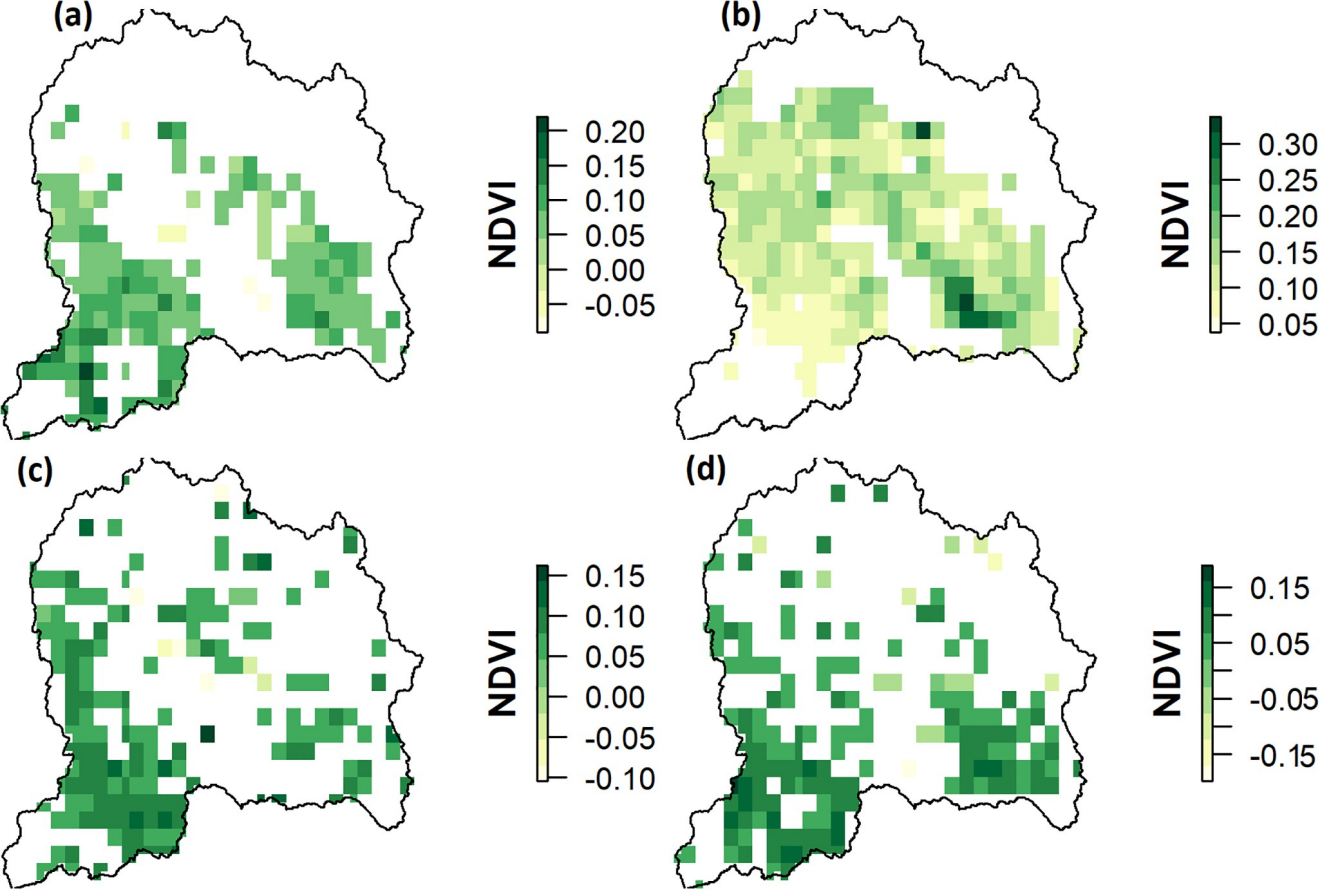
Sen’s slope (p<0.05) in seasonal NDVI during (a) winter (Q1), (b) spring (Q2), (c) summer (Q3) and (d) autumn (Q4) from 1982 to 2015.

On a seasonal basis, pixel-wise trends of NDVI and climatic variables were derived from time-series datasets during each quarter of the year from 1982 to 2013. A significant increase (p<0.05) in NDVI and Tmin was detected and shown in Figs 4 and 5. Similarly, significant trends in Tmax were detected only during summer and autumn (Q3 and Q4). The spatial patterns of NDVI trends were not uniformly distributed over the UJRB and varied across different areas and seasons because of altitudinal variations and discrepancies in responses to climatic variables across vegetation types. Almost all of the UJRB showed a significant increase in NDVI except for some high-altitude areas in the north. In Q1, NDVI increased in the central, eastern and southern regions of the UJRB.

**Fig 6:**
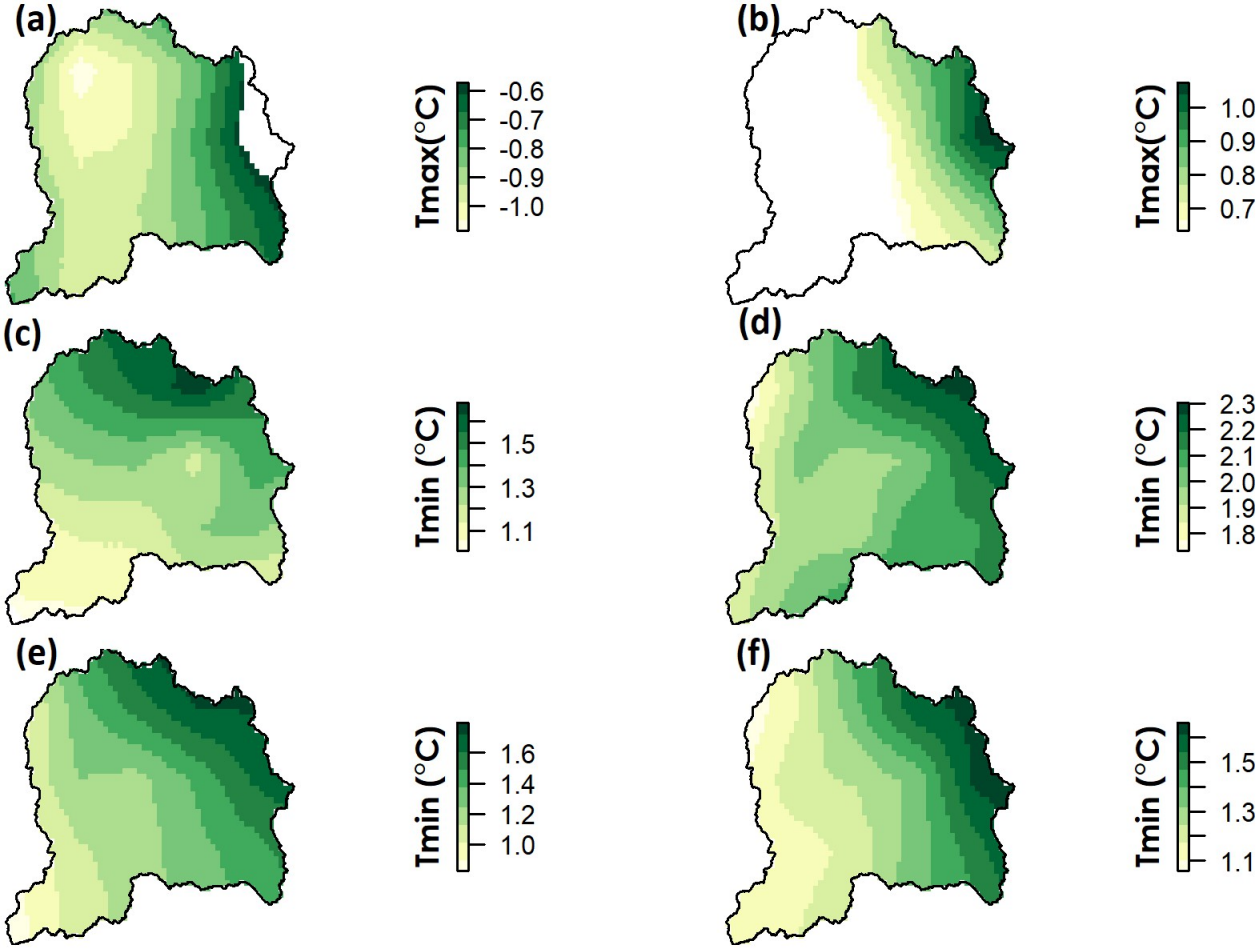
Seasonal trends in Tmax and Tmin over the 34 years period. All p-values greater than 0.05 are masked and only statistically significant (p<0.05) trends are presented. (a) significant trends in Tmax during Q3, (b) significant trends in Tmax during Q4 (c) significant trends in Tmin during Q1 (d) significant trends in Tmin during Q2 (e) significant trends in Tmin during Q3 and (f) significant trends in Tmin during Q4.

The highest increase in NDVI was detected during spring (Q2). The NDVI trends during spring remained positive over the whole of the UJRB compared to other three quarters (Q1, Q2 and Q3) when significant negative trends were also detected in some parts of the UJRB. These results indicate that among four quarters, vegetative productivity remained higher during spring from 1982 to 2015. The seasonal trends in Tmax were found to be significant during summer (Q3) and autumn (Fig 6). During summer, Tmax showed a negative trend over the UJRB, and increased significantly during autumn mainly in the eastern region of the UJRB. Unlike Tmax, Tmin increased significantly (p<0.05) during all four quarters (Fig 6).

### Regional scale annual and seasonal correlation

A correlation analysis of annual NDVI spatially averaged over each LULC of the UJRB showed significant positive correlations with annual Tmin, while weak negative correlations were found with annual river flow (Table 3). NDVI of HV showed significant positive correlation with Tmin and Tmax.

**Table 3:** Annual correlation of NDVI of vegetation classes with precipitation (precip), maximum temperature (tmax) and minimum temperature (tmin) over the UJRB.

Variables	UJRB	CA	EF	MT	Shrubs	HV
Tmax (°C)	0.13	0.05	0.18	0.05	0.07	0.58
Tmin (°C)	0.51	0.51	0.54	0.48	0.39	0.4
Precipitation (mm)	-0.05	0.05	-0.08	-0.03	0	-0.36
River Flow (*m*^3^/*s*)	-0.2	-0.19	-0.26	-0.22	-0.12	-0.26

A correlation coefficient of 0.3 corresponds to p<0.05, 0.4 corresponds to p<0.01 and 0.5 corresponds to p<0.05.

A stronger correlation was detected among seasonal metrics of the NDVI from vegetation types and hydroclimatic variables compared to annual metrics (Table 5). Seasonal precipitation during the summer showed a strong positive correlation with the NDVI of all vegetation types in the following season (e.g., autumn). This correlation was the strongest with CA while the weakest with HV. Generally, river flow showed significant negative correlation with NDVI of all vegetation types. A highly significant negative correlation of river flow with NDVI of HV during winter (Q1) and spring (Q2) was detected.

A stronger correlation between NDVI and Tmin for all vegetation types except HV compared to Tmax was detected. However, a highly significant positive correlation of NDVI of HV with Tmin and Tmax was found during spring (p<0.05). Summer (Q3) Tmin showed strong positive correlation with NDVI for all vegetation types during all seasons. In contrast to Tmin, Tmax during summer generally showed negative correlation with NDVI for all vegetation types except HV.

### Pixel scale annual and seasonal correlation

**Fig 7:**
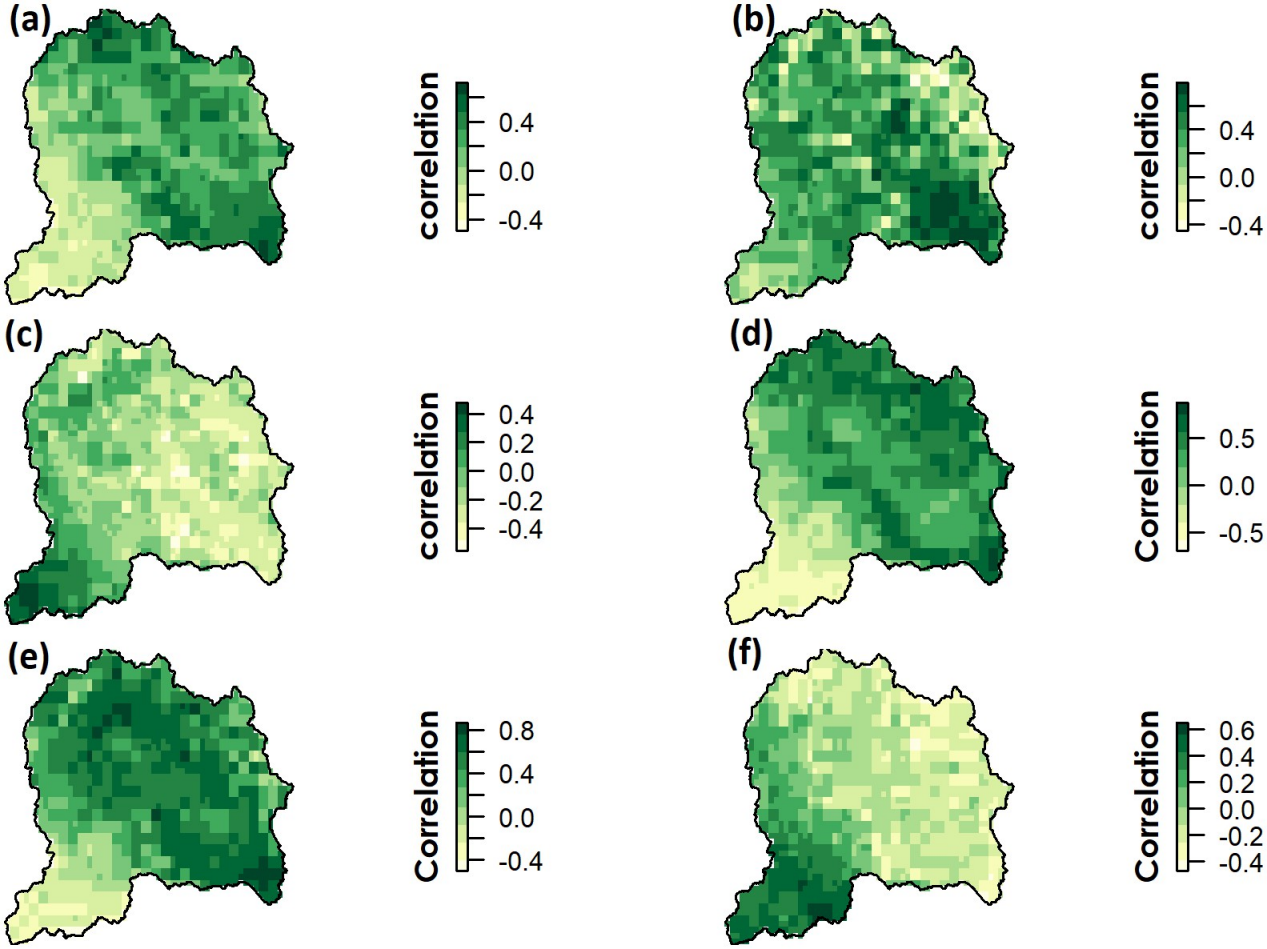
Annual correlation coefficient of NDVI with Tmax (a) Tmin (b) and precipitation (c). Similarly, correlation coefficient of NDVI with Tmax (d) Tmin (e) and Precipitation (f) in spring (Q2) over the UJRB during 1982–2013.

The spatial variability of annual correlation coefficient values between NDVI and climate variables are shown in Fig 7. Only a smaller region in the south of the UJRB, which is mainly a plain area, showed a significant (p<0.05) positive correlation between NDVI with precipitation, while a major part in the northern and central uplands showed negative correlation. It is noteworthy that, although annual NDVI was negatively correlated with precipitation, the latter still played a significant role in vegetation growth in some specific areas, denoted by significant correlations between NDVI and precipitation (Fig 7).

**Fig 8:**
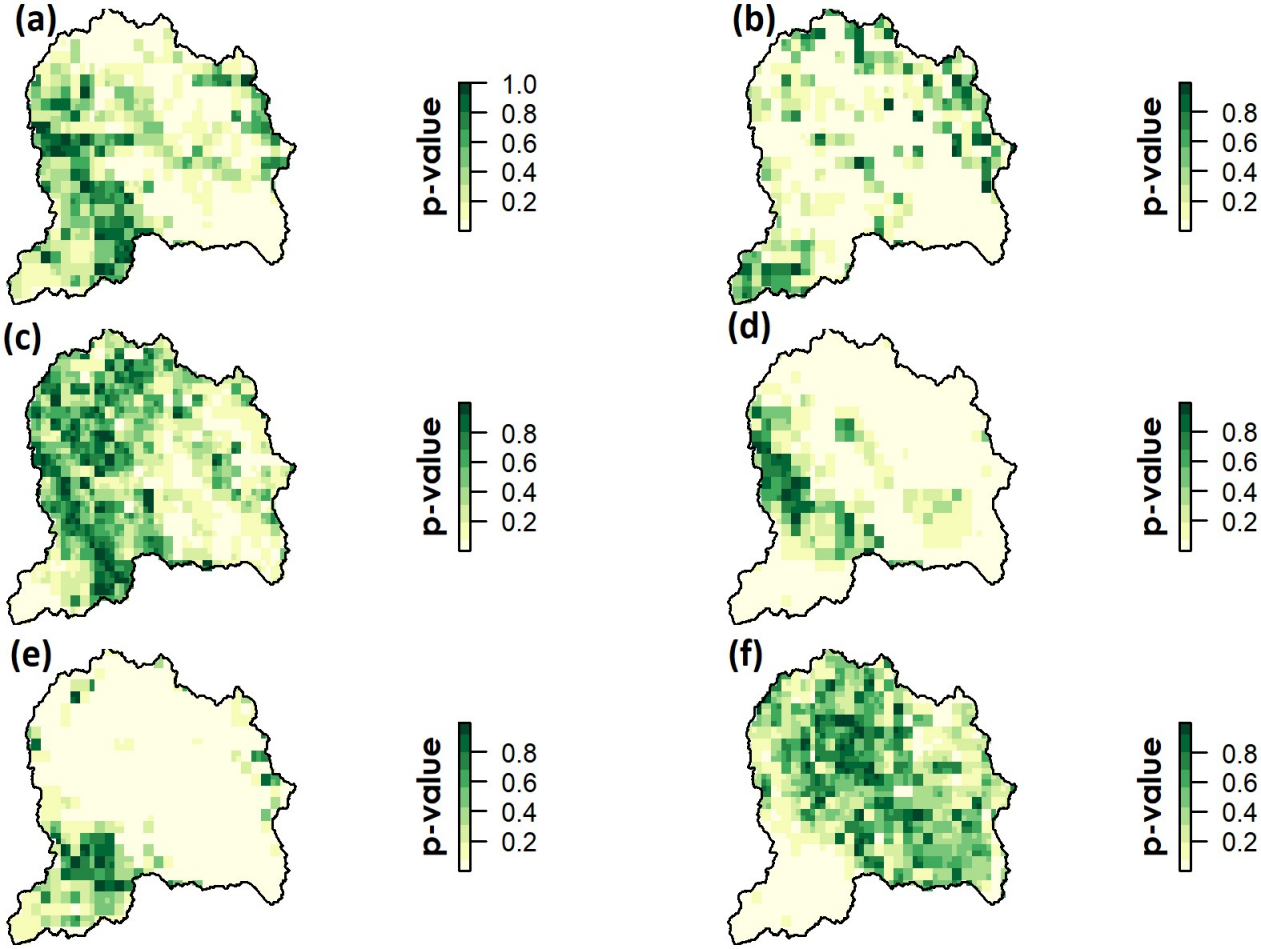
Annual correlation coefficient represented by p-values of NDVI with Tmax (a), Tmin (b) and precipitation (c). Similarly, seasonal correlation coefficient of NDVI with Tmax (d) Tmin (e) and Precipitation (f) in spring (Q2) over the UJRB during 1982–2013.

A strong positive correlation of NDVI with Tmax and Tmin was observed mainly in the central and northern regions of the UJRB (Fig 7). The spatial variability of NDVI correlation with climate variables showed that much of the UJRB had a significant correlation with Tmin, whereas a correlation of NDVI with Tmax and precipitation was found in smaller areas. Seasonal pixel-wise correlation of NDVI with climatic variables was found to be significant over the UJRB mainly during spring (Fig 8), but was not uniform across the UJRB. NDVI was found to be significantly correlated with precipitation only during spring, where NDVI was mostly negatively correlated in the northern region while positively correlated in the southern region of the UJRB (Fig 8).

Similarly, seasonal spatial patterns of correlation between NDVI and Tmax were found to be significant (p<0.05) during the spring. NDVI showed significant positive correlation with Tmin over much of the UJRB except southern region. NDVI correlation with Tmax and Tmin were also significant over much of the UJRB during spring only.

## Discussion

The NDVI of all vegetation types except HV increased considerably both annually and seasonally, over most of the UJRB (Figs 4 and 5 and Table 2). These results indicate a greening trend across the UJRB over the past 34 years. This is consistent with results from previous studies over the Tibetan Plateau (e.g., [53, 54, 68]). However, in the UJRB, increasing trends in NDVI are not consistent across the time series and greater increases are mostly observed during the 1980s and 1990s. Since then, the NDVI of most vegetation types shows a stable or even decreasing trend (1995 to 2003; Fig 2). Similarly, interannual changes in NDVI of HV over the Tibetan Plateau show that greening trends mainly occurred during 1980s and 1990s [68, 69] and negative trends in NDVI are reported from 2000s onward. In other parts of the World, such as Eurasia, increasing trends in NDVI are also reported during 1980s and 1990s but has marginally decreased during during the period 1997 to 2006 [70, 71].

Interannual NDVI of the HV showed a significant decrease during 2000 to 2015 (Fig 2). Permafrost zones in alpine cold ecosystems are sensitive to climate change and any alterations in the permafrost can significantly affect alpine ecosystems. In the UJRB, HV mainly lies in the permafrost zone (Fig 1). According to [72], alpine cold meadow ecosystems in the Tibetan Plateau under climate warming conditions will undergo serious degradation over the next 50 years if warming increases as predicted. [73] linked the marked thawing, thinning, and degradation of permafrost due to warming across the Tibetan Plateau. Therefore, degradation of permafrost will result in a decrease in vegetation and accompanied drying of the soil surface. Similarly, the NDVI of HV showed decreasing trends during the winter (Table 4), which is in line with a reduction of vegetation in the permafrost across Tibetan plateau [73]. However, a reduction in HV in the UJRB could be related to an increase in anthropogenic activity.

**Table 4:** Seasonal Mann-Kendall trend statistics calculated from spatially averaged data of precipitation, maximum temperature (Tmax) and minimum temperature (Tmin) over the whole UJRB and river flow in four quarters (Q1, Q2, Q3 and Q4) during 1982 to 2015.

Variables	Quarters	p-value	z-value	Sen’s Slope
Precipitation (mm)	Q1	0.1	1.7	0.34
	Q2	0.7	0.45	0.066
	Q3	<0.05	2.1	0.34
	Q4	<0.05	-2.2	-0.36
Tmax (°C)	Q1	<0.05	2.8	0.024
	Q2	<0.05	2.2	0.018
	Q3	<0.05	-7.6	-0.023
	Q4	<0.05	4.7	0.018
Tmin (°C)	Q1	<0.05	4.8	0.038
	Q2	<0.05	6.8	0.057
	Q3	<0.05	11	0.041
	Q4	<0.05	8	0.038
River Flow (*m*^3^/*s*)	Q1	0.4	0.9	0.88
	Q2	<0.05	-3.9	-22
	Q3	0.9	0.2	0.5
	Q4	0.1	1.5	1.7

### NDVI and precipitation

The greater spatial variability in NDVI trends across the UJRB are due to variation in responses of vegetation classes to climatic variables. Plant growth is influenced by changes in soil moisture and local water balance, which in turn are dependent on climatic variables (e.g., precipitation and temperature). This is reflected in the results in the form of greater spatial variability in correlations of annual and seasonal NDVI with precipitation across the UJRB (Figs 7 and 8). NDVI correlations with precipitation over the UJRB differ with vegetation classes. For instance, the NDVI has been positively correlated with precipitation in the southern region of the UJRB, while precipitation shows no correlation and weak negative correlation in the northern parts of the UJRB (Fig 7). According to [74], the growth of some forest types in the central Himalaya are invariant to short term changes in precipitation due to the abundance of moisture available from the Arabian sea and Bay of Bengal during summer. Here, the NDVI showed weak correlations with precipitation over much of the UJRB in the northern high-altitude region. Similar trends are detected in high-latitude systems of north western Canada, where long-term NDVI was negatively correlated with precipitation [75]. Similarly, [75] found a weak negative relationship between NDVI and precipitation for temperature classes below 12°C, and positive relationships for warmer temperature classes. An increase in precipitation generally leads to a decrease in sunshine hours [76], which results in a negative correlation between NDVI and precipitation. In this study, NDVI correlations with climatic variables are consistent with [74] where they found significant correlations of NDVI with temperature data compared to precipitation data in the Central Himalayas.

### NDVI and temperature

Annual NDVI is highly correlated with Tmin than Tmax across UJRB (Fig 7). During spring, NDVI shows a significant correlation with Tmin and Tmax, which varied greatly across the UJRB(Fig 8). The NDVI for all vegetation types was positively correlated with Tmin during the spring, which indicates that increases in temperature could strengthen vegetation growth due to a lengthening of the growing season. Similar increases in NDVI for all vegetation classes due to higher seasonal temperature are also reported in previous studies [5, 53, 77]. However, responses of NDVI to warming differ in the northern high-altitude region compared to the southern low-altitude region of the UJRB (Fig 7). For example, significant positive correlations in the northern UJRB indicate strong responses of NDVI to increasing Tmin and Tmax, while weaker responses of NDVI to Tmin and Tmax are prevalent in the southern, low-altitude areas of the UJRB. These results show that the correlation of NDVI with climatic variables change rapidly with altitudinal shift over the UJRB. These results are consistent with [78] in the Qilian Mountains of North-western China, where they observed that NDVI was controlled by precipitation and air temperature at low elevations, while increases in NDVI were mainly controlled by air temperature alone at high elevations. Warming during the spring (Q2) results in a significant increase in NDVI (Table 4), which is reported in the western Himalaya [79] or neighbouring Tibet [42, 80]. These results indicate that temperature is still a limiting factor for vegetation growth at high elevation sites. These results confirm the findings of vegetative vigour with maximum increased temperature in the central Himalaya [74]. However, a weak correlation with NDVI in the southern region of the UJRB may be related to the increased daytime temperature (Tmax) that results in increased evapotranspiration and reduced soil moisture. These weaker NDVI responses to warming because of differences in altitude and environment are reported in previous studies in other parts of the world (e.g., [53, 70]).

The increase in Tmin (1.54°C) was much higher than Tmax (0.37°C) over 34 years in the UJRB (Table 4). The asymmetric annual and seasonal daytime and night-time warming affects plant photosynthetic activity and yield in different ways [25, 81–83]. The dominant role of increasing Tmin (e.g., night time warming) on NDVI indicates that ecosystems of the UJRB would be most affected by climatic warming. However, the responses of NDVI to daytime and night-time temperature are uncertain in global studies. For instance, increases in Tmax are positively correlated with NDVI in wetter and colder regions and negatively correlated in arid and semi-arid regions, where increase in Tmin suppresses vegetation growth [84]. In contrast, increases in Tmin are positively correlated with vegetation in the high cold steppe and meadow steppe zones which is in line with a greening trend mainly controlled by increases in Tmin over the UJRB. However, increases in Tmax are positively correlated with NDVI of wetter and colder regions of the Tibetan Plateau [83] compared to Arctic ecosystems where greening is controlled mainly by Tmax (i.e., daytime warming). In the summer, Tmax decreased significantly but NDVI showed a significant increase during summer (Table 4). According to [85], the smaller response of NDVI to Tmax during the summer is due to the saturation of the NDVI over dense vegetation. Similar phenomena are also observed on the Tibetan Plateau where Tmin has a more dominant enhancing effect on ecosystems than Tmax [54]. In contrast to the well-defined relationship of NDVI for all vegetation classes with Tmin, only the NDVI of HV shows a significant positive correlation with annual Tmax (Table 3). This may be related to plant available water capacity and Tmax effect on vegetation growth [53]. It is well established that HV has lower evapotranspiration than forested vegetation [86]. It is suggested that vegetation growth of CA, EF, SH and MT is limited due to compounding effects of low water availability and increase in Tmax [68, 87]. Therefore, the precipitation during the summer leads to significant increases in NDVI of EF and CA in the following season (e.g., autumn; Table 4).

In the southern areas of the UJRB, which are mostly occupied by cultivated agriculture (CA), mixed trees (MT) and shrubs (SH), higher Tmax and lower precipitation is dominant (Fig 3). The area can be characterized as a semi-arid region. Here, daytime warming can lead to accelerated leaf transpiration and higher ET, reduced soil moisture and exposure to water stress which suppresses vegetation growth and photosynthesis. Therefore, weak or no correlations between Tmax and NDVI were found in the southern region of the UJRB (Fig 7). In contrast to the southern region, increase in Tmax effectively increased the vegetation growth in the northern and central eastern semi-humid regions where mean summer Tmax remained below 10°C with excess soil moisture. In the southern region, significant negative correlations while in the northern region significant positive correlations between NDVI and Tmax (Fig 7) indicate the role of water availability and water stressed conditions affecting vegetation growth during the spring season. According to [84], an increase in vegetation growth with increasing Tmax was found in the cold and humid regions, but no correlation in arid and semi-arid regions. These results are consistent with the research findings in the UJRB.

Tmin shows a strong positive correlation with NDVI during all seasons, whereas Tmax shows a negative correlation with NDVI for all vegetation classes during summer (Table 5). Significant negative correlations between the NDVI and Tmax during the summer suggest that increasing NDVI for all vegetation types may result in a decrease in Tmax with higher evapotranspiration rates. Negative correlations between NDVI and Tmax are more consistent and stronger during summer (Q3) than other seasons when vegetation activity is higher, and radiation is more intense. This negative correlation between Tmax with NDVI during summer suggests an evapotranspiration-induced cooling effect during daytime in the UJRB. In the UJRB, much of the central and southern region is intensively cultivated land (Fig 1) which may have a cooling and wetting effect regionally by increasing evapotranspiration as shown by studies with global and regional circulation models [88–90]. According to [91], vegetation greening and evapotranspiration have a strong cooling effect on local temperatures. A similar response of ET induced cooling has been noted over the Tibetan Plateau [68]. According to [68], the cooling effect during summer is in response to vegetation greening and is likely to reduce the daytime temperature (Tmax) rather than night-time (Tmin). They found that vegetation greening over central Asia plays a significant role in cooling effects on the local temperatures in arid, semi-arid and semi-humid regions, in contrast to humid regions, where albedo suppresses this. According to [88], intensification of agriculture in Higher Mountain Asia (HMA) may have a profound impact on summer temperatures locally by reducing net radiation and changes in summer snowfall on Kunlun Shan. Recent research shows a vegetation greening trend along with enhanced evapotranspiration over the past three decades in the Tibetan Plateau [5, 32]. In the context of the above discussion, it is more likely that enhanced vegetation growth in the UJRB has led to an increase in evapotranspiration and evaporative cooling during the summer.

**Table 5:** Seasonal correlation of NDVI of vegetation classes with climatic variables in each quarter over the UJRB.

NDVI	River Flow (*m*^3^/*s*)					Precipitation (mm)				Tmin (°C)				Tmax (°C)			
Evergreen forest		Q1	Q2	Q3	Q4	Q1	Q2	Q3	Q4	Q1	Q2	Q3	Q4	Q1	Q2	Q3	Q4
	Q1	-0.03	**-0.43**	**-0.4**	-0.18	0.04	-0.21	-0.07	-0.08	0.15	**0.38**	**0.43**	0.25	0.31	0.26	-0.04	0.25
	Q2	0.07	**-0.4**	-0.1	0.03	0.12	0.04	0.17	-0.25	0.23	**0.56**	**0.55**	**0.46**	0.25	0.28	**-0.4**	**0.39**
	Q3	0.21	-0.17	0	0.07	0.19	0.1	0	-0.12	0.04	0.06	**0.41**	0.05	-0.06	-0.29	-0.17	-0.02
	Q4	0.34	-0.15	0.03	-0.02	0.14	0.02	0.22	-0.2	**0.5**	**0.4**	**0.48**	0.23	0.21	0.08	-0.28	**0.44**
Shrubs	Q1	-0.15	-0.33	-0.19	-0.05	0.06	-0.17	-0.02	-0.09	-0.07	0.24	**0.43**	0.15	0.07	0.09	0.07	0.05
	Q2	0.06	-0.24	0	0.07	0.15	0.13	0.12	-0.23	0.05	**0.4**	0.5	**0.41**	0.1	0.17	-0.24	0.22
	Q3	0.24	-0.14	-0.03	-0.08	0.41	-0.02	0.12	-0.16	0.15	0.22	**0.45**	0.1	-0.04	-0.08	-0.08	0.13
	Q4	0.25	0.06	0.26	0.08	0.11	0.08	0.41	-0.17	0.33	0.21	0.2	-0.03	0.11	-0.01	-0.33	0.23
Cultivated Agriculture	Q1	**0.4**	-0.33	-0.32	-0.14	0.27	-0.06	-0.23	-0.26	0.21	**0.4**	**0.61**	0.25	0.16	0.12	-0.05	0.16
	Q2	0.31	-0.15	-0.03	0.06	0.4	0.26	0.07	-0.25	0.02	0.25	**0.56**	0.34	-0.06	-0.11	-0.26	0.17
	Q3	0.09	-0.2	0.1	0.07	0.32	-0.1	0.27	-0.15	0.13	**0.42**	**0.44**	0.18	-0.03	0.13	-0.2	0.19
	Q4	0.02	-0.31	0.22	0.4	0.08	0.05	**0.49**	-0.01	0.4	**0.4**	**0.5**	0.28	0.16	0.09	**-0.4**	0.17
Mixed Trees	Q1	0.23	-0.28	-0.32	-0.19	0.26	-0.07	-0.09	-0.14	0.18	0.34	**0.53**	0.3	0.09	0.06	-0.14	0.25
	Q2	0.18	-0.19	-0.06	0.05	0.24	0.23	0.06	-0.2	0.04	0.27	**0.54**	0.36	0.07	-0.04	-0.26	0.21
	Q3	0.14	-0.28	-0.13	-0.02	0.23	0.06	-0.01	-0.19	0.06	0.25	**0.41**	0.24	-0.01	-0.09	-0.22	0.17
	Q4	0.34	-0.19	0.05	0.1	0.14	0.01	0.19	-0.12	0.51	**0.4**	**0.5**	0.23	0.18	0.05	-0.34	0.33
Herbaceous Vegetation	Q1	-0.61	-0.25	0.02	0.04	-0.31	-0.05	0.02	0.13	-0.19	-0.01	-0.21	0.01	0.23	0.2	0.13	-0.14
	Q2	-0.35	-0.54	-0.22	-0.15	-0.34	-0.34	0.11	-0.29	**0.4**	**0.78**	0.28	**0.52**	**0.52**	**0.78**	-0.22	**0.56**
	Q3	0.25	0.03	0.02	-0.01	0.13	0.07	-0.06	0.1	-0.05	-0.17	0.06	-0.26	-0.09	**-0.4**	0.06	-0.16
	Q4	0.11	0.26	0.04	**-0.4**	0.05	-0.09	0.16	-0.32	0.25	0.14	-0.1	-0.13	0.18	0.18	-0.06	**0.41**
All Vegetation Types	Q1	0.1	**-0.4**	-0.23	-0.1	0.12	-0.19	-0.1	-0.19	0.28	**0.41**	**0.48**	0.22	0.33	0.23	-0.04	0.21
	Q2	0.14	**-0.4**	-0.05	0.09	0.15	0.16	0.09	-0.22	**0.44**	**0.51**	**0.59**	**0.45**	0.33	0.18	**-0.4**	0.27
	Q3	0.25	-0.07	0.06	0.07	0.25	0.01	0.01	-0.02	-0.08	-0.03	0.27	-0.06	-0.11	-0.28	0.01	-0.07
	Q4	0.19	0.02	0.15	-0.06	0.14	0.08	0.31	-0.14	0.32	0.2	0.34	0.09	0.07	-0.07	-0.17	**0.4**

A correlation coefficient of 0.3 corresponds to p<0.05, 0.4 corresponds to p<0.01 (bold) and 0.5 corresponds to p<0.001.

### NDVI and hydrology

The spatiotemporal changes in climate, vegetation and their linkages with hydrology are also investigated in the UJRB. In annual long-term trends of river flow and NDVI, both variables show significant opposing trends over the 34-year study period (Table 1). However, seasonal trends show that river flow decreased significantly during the spring only, while the NDVI for all vegetation types increased significantly during all seasons. The river flow from the UJRB shows a significant decrease over the period during spring (growing season) where long-term records of climate suggested a noticeable warming trend (1.28°C) (Table 1). Transpiration rates increase with an increase in temperature, especially during the growing season, due to greater release of water to the atmosphere. In the UJRB, spring peak flow is driven mainly from melting snowpack ([16]; Table 4). However, the strong increase in NDVI and temperature during Spring may also contribute to reduced river flows, alongside changes in the mountain cryosphere, because of enhanced transpiration and evapotranspiration rates as a consequence of higher temperatures across the UJRB.

The effect of climate change on hydrology can be better explained by an understanding of evapotranspiration. It is well-known that enhanced vegetation growth is linearly correlated with evapotranspiration [55, 92]. [93] quantified the sensitivity of vegetation and evapotranspiration in the cold-limited Upper Kings River basin, California. The results show that warming has the potential to reduce surface water supply by upland vegetation expansion activity, density or coverage. According to [94], climate variability is a major driving factor for decrease in streamflow in the Taolinkou catchment in China. In other studies, the impact of vegetation on evapotranspiration and a reduction in streamflow has been noted and suggested this is accompanied by increased canopy interception and evapotranspiration due to enhanced vegetation cover [57, 94, 95].

However, some studies show a negative relationship between evapotranspiration and runoff [96]. Whilst the effect of evapotranspiration and vegetation cover on runoff has been well researched using in situ field experiments, monitoring at catchment scales with remote sensing and parametrization within hydrological models is still relatively poorly understood. In the UJRB, enhanced vegetation growth especially during the greening phase (e.g., spring) resulted in an increase of evapotranspiration which may be attributed as a major driving factor behind significant reduction in runoff during spring. [97] studied the reduction of spring flow through quantification of precipitation and evapotranspiration in the Yellow River of northeastern Tibetan Plateau. They observed that a decrease in spring flow reduction was due to a decrease in precipitation (70%) and increase in evapotranspiration (30%) during the 1990s. However, a reduction in spring flow resulted from just a 3% decrease in precipitation while 97% was accounted for through increased evapotranspiration in the 2000s. The widespread increase in vegetation growth and warming during all seasons favour the increase in evapotranspiration in the UJRB. The significant decrease in river flow in the UJRB may be attributed to enhanced vegetation growth, which resulted in higher evapotranspiration rates due to climate warming. Reduction in river flow is also likely to be linked to a significant (p<0.05) decrease in winter snowpack and snowmelt during spring ([16]; Table 4). The high altitude regions contribute substantially to spring flow in the form of snow- and glacier melt during spring [98], but there has been rapid deglaciation in recent decades which is likely to affect the magnitude and timing of such flow [98–100]. The average annual discharge was significantly decreased from the UJRB during 1962 to 2018 [99, 100]. Overall, increasing trends of temperature, decrease in precipitation, snow cover area and river flow, deglaciation and disintegration of glaciers were observed from the glaciered sub-basins of the UJRB [98, 99]. Our analysis suggests that decrease in river flow could be attributed to cumulative effect of climate variablity, enhanced vegetation growth and warming during spring where evapotranspiration might have played a significant role alongside changes to the mountain cryosphere. However, further research is needed to explore the relative roles of changes in evapotranspiration, snowpack and glacier cover in the reduction of spring flow from the UJRB.

## Conclusion

This study has presented the results from analysis of long-term changes (1982–2015) in vegetation productivity and its relationship with hydrometeorological variables in the UJRB and can be concluded as follows:

1. In the UJRB, night-time temperature (Tmin) shows a significant increase during all seasons, whereas day-time temperature (Tmax) shows a significant decrease only in summer.
2. In the UJRB, enhanced vegetation growth is attributed to increase in night-time warming especially during spring in contrast to daytime temperature which only contributed to significant increase in NDVI of herbaceous vegetation.
3. A significant decrease in summer temperature (local cooling effect) may be attributed to higher vegetation growth and enhanced evapotranspiration in the UJRB.
4. Warming during spring in the UJRB has potentially reduced the river flow by accelerating the vegetative growth. This decrease in spring flow from the UJRB shows a major shift in seasonal redistribution of hydrology in the UJRB. Furthermore, enhanced vegetation growth results in evaporative cooling which results in decrease of summer daytime temperature.
5. At low altitudes, vegetation growth suffers greatly due to water stress but flourishes at high altitudes with increasing air temperature due to increases in soil moisture by melting of snow. It is crucial to understand that climate warming may affect ecology and hydrology at high altitudes of the UJRB.

Overall, the interplay between vegetation, atmospheric processes and river flow has resulted in an increase of NDVI, summer cooling and reduced river discharge during the growing season. In this study, it is evident that night-time temperature acts as a major driving factor behind vegetation growth and its ecosystem carbon sink function over the UJRB. However, a declining trend in river flow at Mangla Dam outlet is alarming because it will reduce water supply for agriculture, industrial and municipal uses. Due to limitations of this study, changes to snow and glacier cover were not included in this analysis, however we have included further information on the role of glacier melt and changing extent to provide further context. A significant decrease in the Jhelum river flow at Mangla, especially during spring, needs further research to explore the relative effects of changes in snowpack and glacier ice cover versus evapotranspiration in reduction of spring river flows from the UJRB over the study period.

## Supporting information

S1 Fig Monthly precipitation. Average monthly precipitation over the UJRB during 1982 to 2015.
(TIFF)

S2 Fig Yearly precipitation. Average annual precipitation over the UJRB during 1982 to 2015.
(TIFF)

S3 Fig Monthly minimum temperature. Average monthly Tmin over the UJRB during 1982 to 2015.
(TIFF)

S4 Fig Yearly minimum temperature. Average annual Tmin over the UJRB during 1982 to 2015.
(TIFF)

S5 Fig Monthly maximum temperature. Average monthly Tmax over the UJRB during 1982 to 2015.
(TIFF)

S6 Fig Yearly mamixum temperature. Average annual Tmax over the UJRB during 1982 to 2015.
(TIFF)

S7 Fig Monthly NDVI. Average monthly NDVI over the UJRB during 1982 to 2015.
(TIFF)

S8 Fig Yearly NDVI. Average annual NDVI over the UJRB during 1982 to 2015.
(TIFF)
