# Supplementary figures and images for: Assessment of climate change effects on vegetation and river hydrology in a semi-arid river basin
Source: PLoS One. 2022 Aug 29;17(8):e0271991. doi: 10.1371/journal.pone.0271991 (PMC9423654; doi:10.1371/journal.pone.0271991)

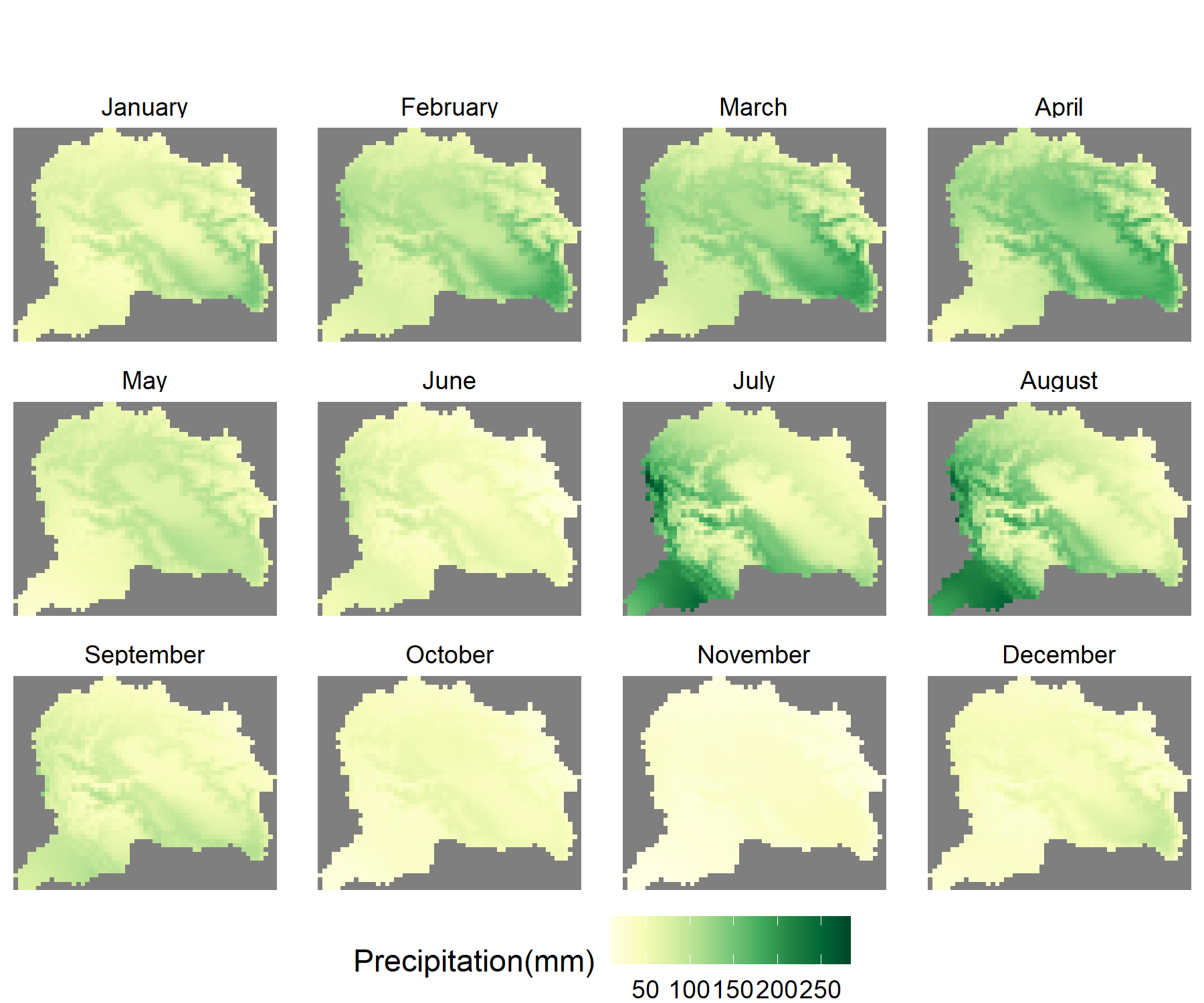

Supplement: S1 Fig — Average monthly precipitation over the UJRB during 1982 to 2015. (TIFF) [file pone.0271991.s001.tiff]

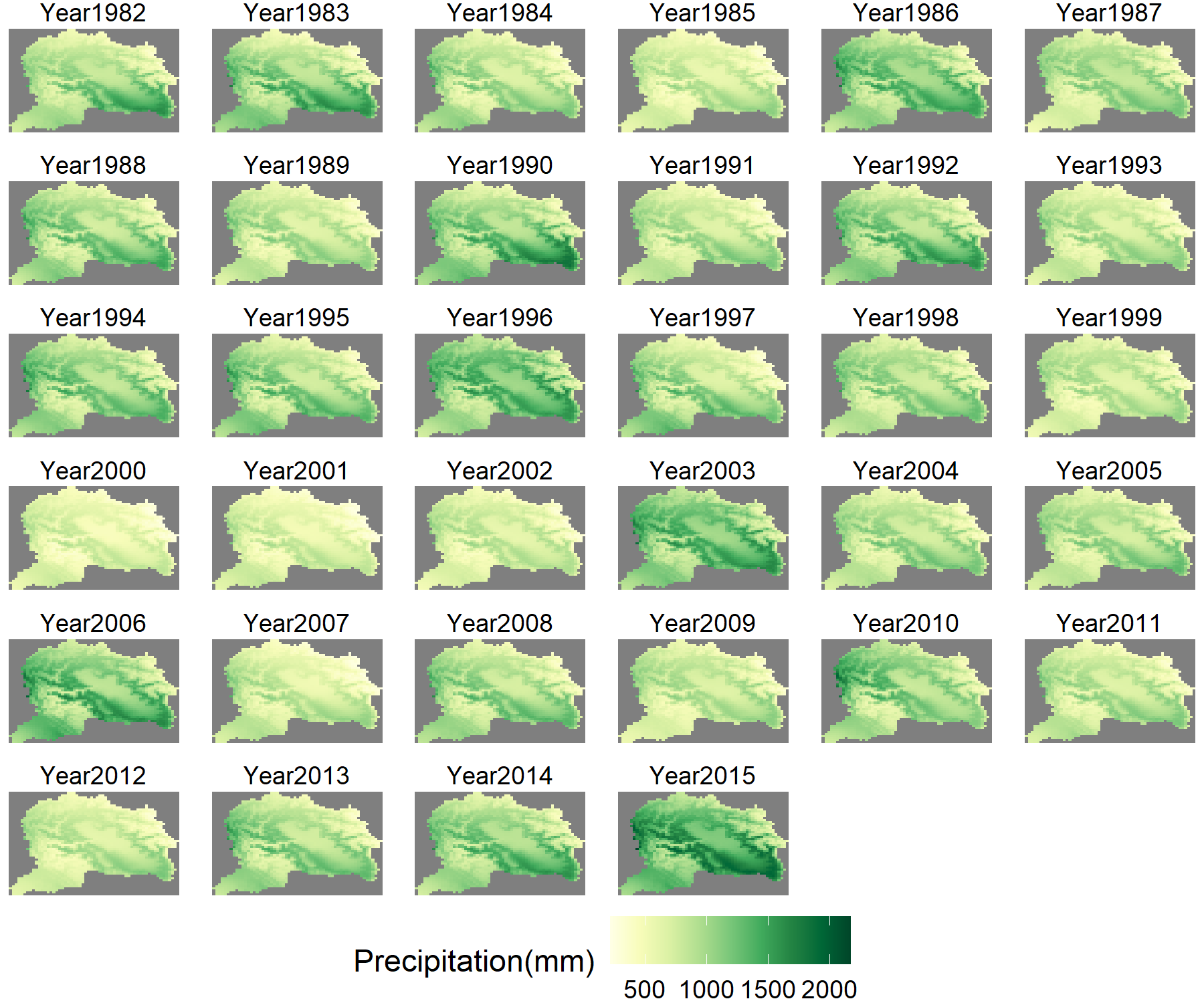

Supplement: S2 Fig — Average annual precipitation over the UJRB during 1982 to 2015. (TIFF) [file pone.0271991.s002.tiff]

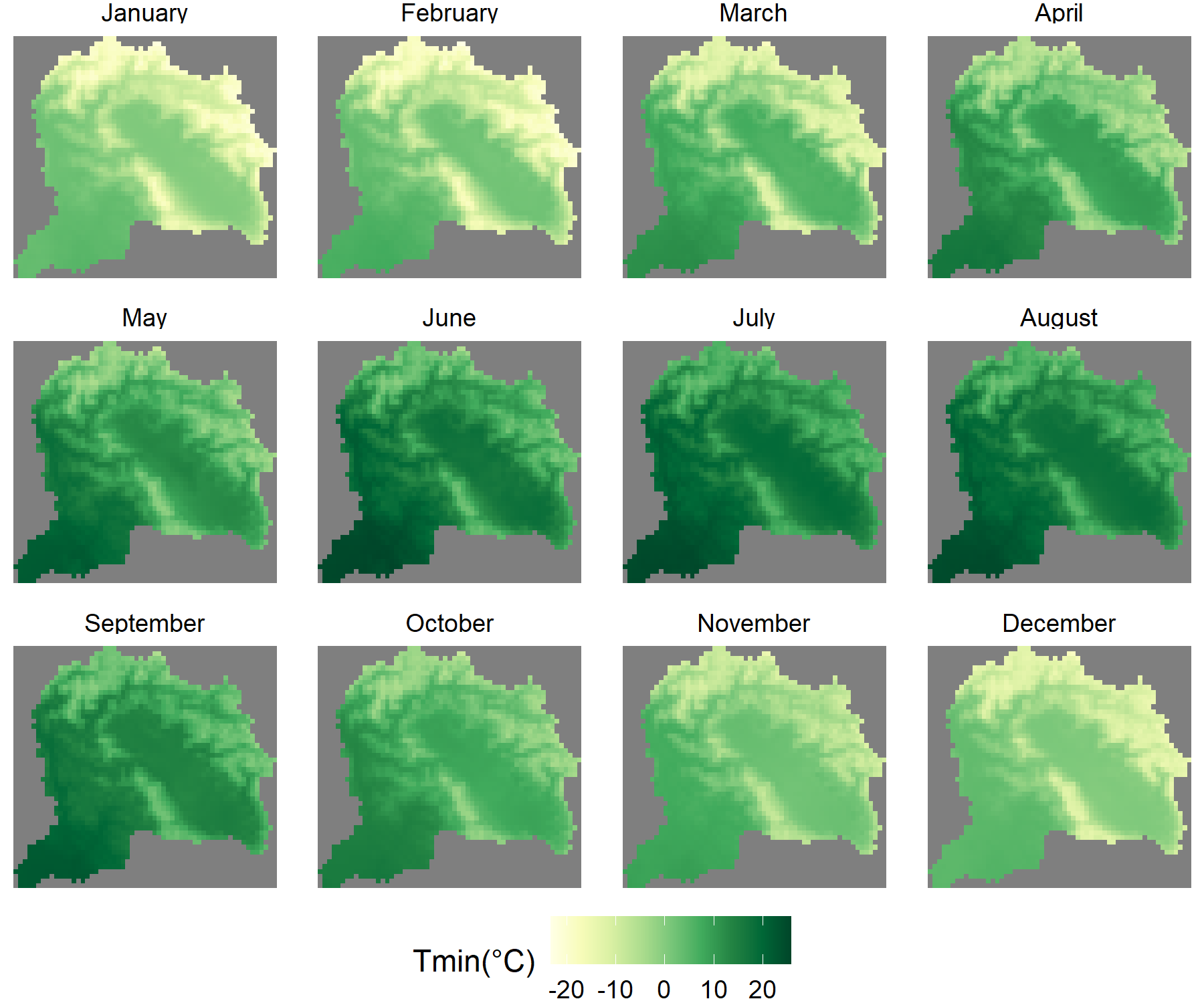

Supplement: S3 Fig — Average monthly Tmin over the UJRB during 1982 to 2015. (TIFF) [file pone.0271991.s003.tiff]

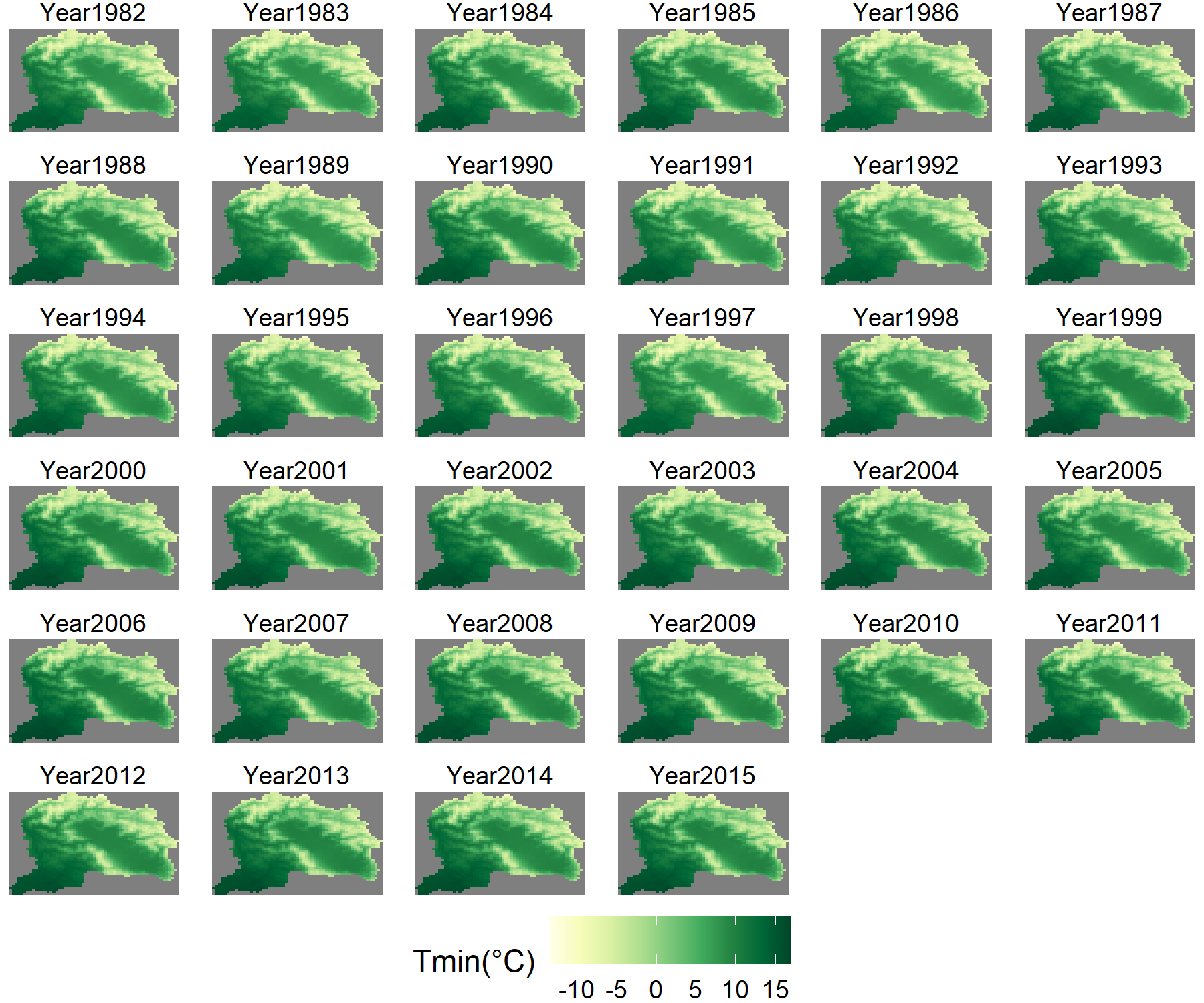

Supplement: S4 Fig — Average annual Tmin over the UJRB during 1982 to 2015. (TIFF) [file pone.0271991.s004.tiff]

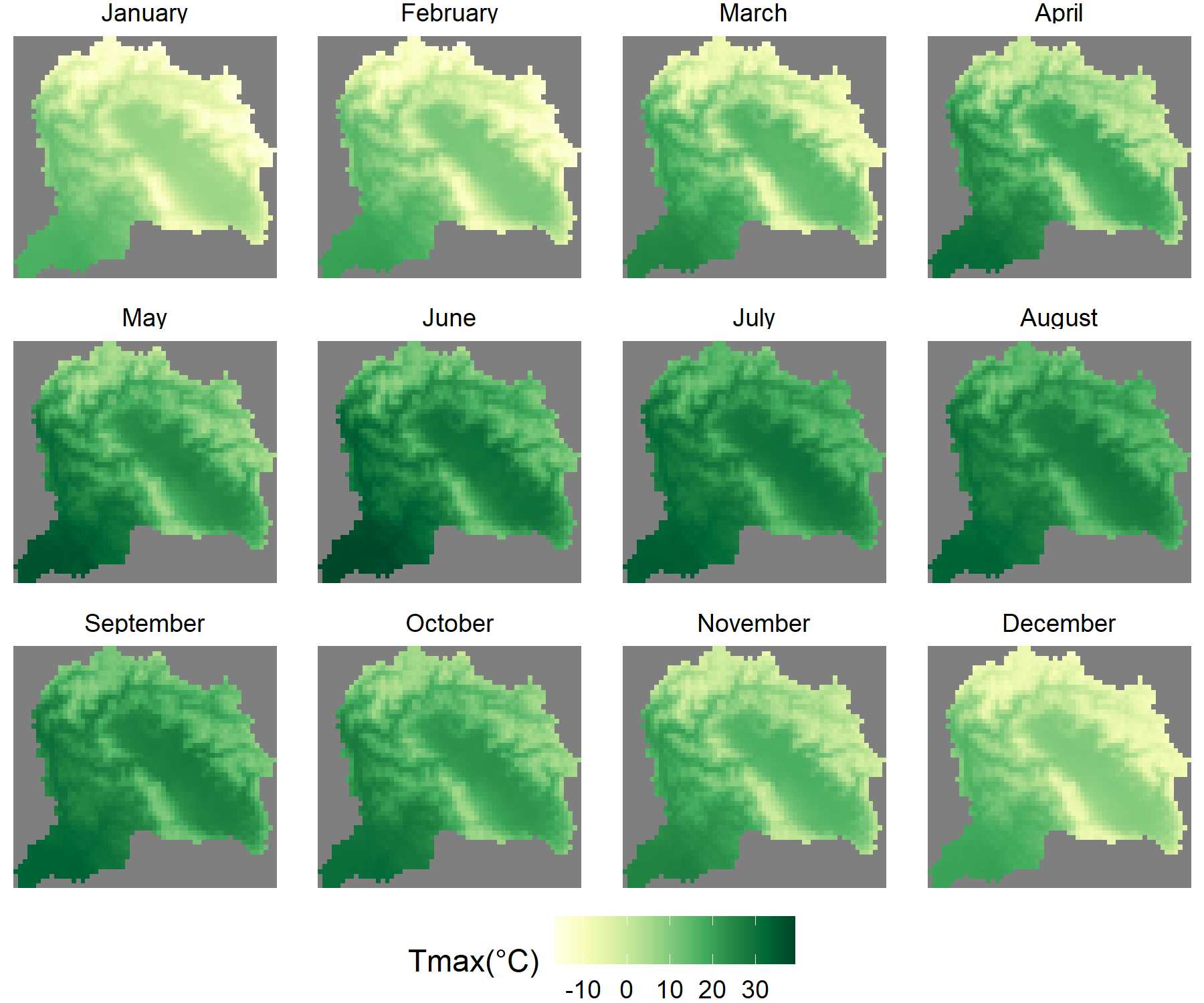

Supplement: S5 Fig — Average monthly Tmax over the UJRB during 1982 to 2015. (TIFF) [file pone.0271991.s005.tiff]

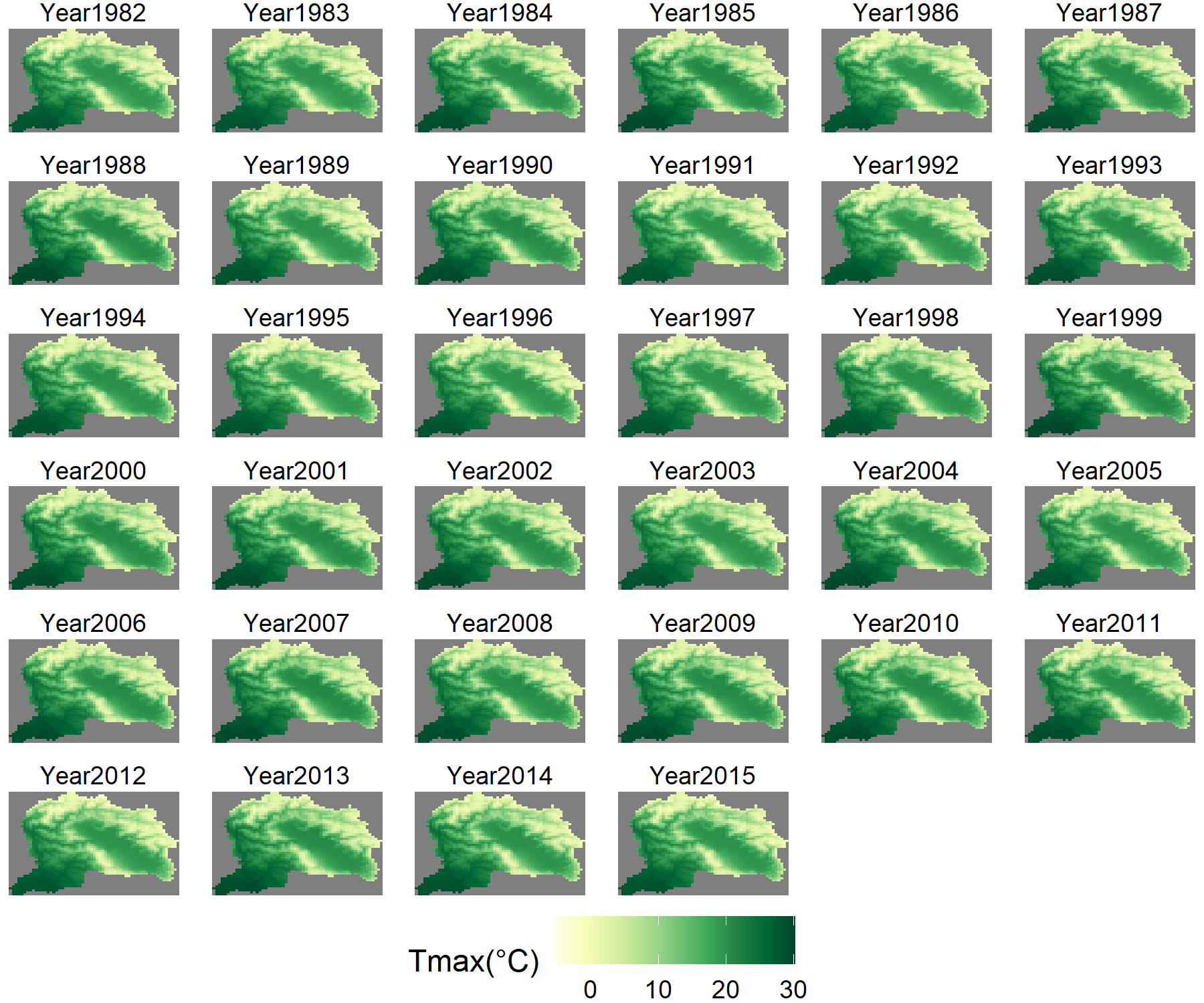

Supplement: S6 Fig — Average annual Tmax over the UJRB during 1982 to 2015. (TIFF) [file pone.0271991.s006.tiff]

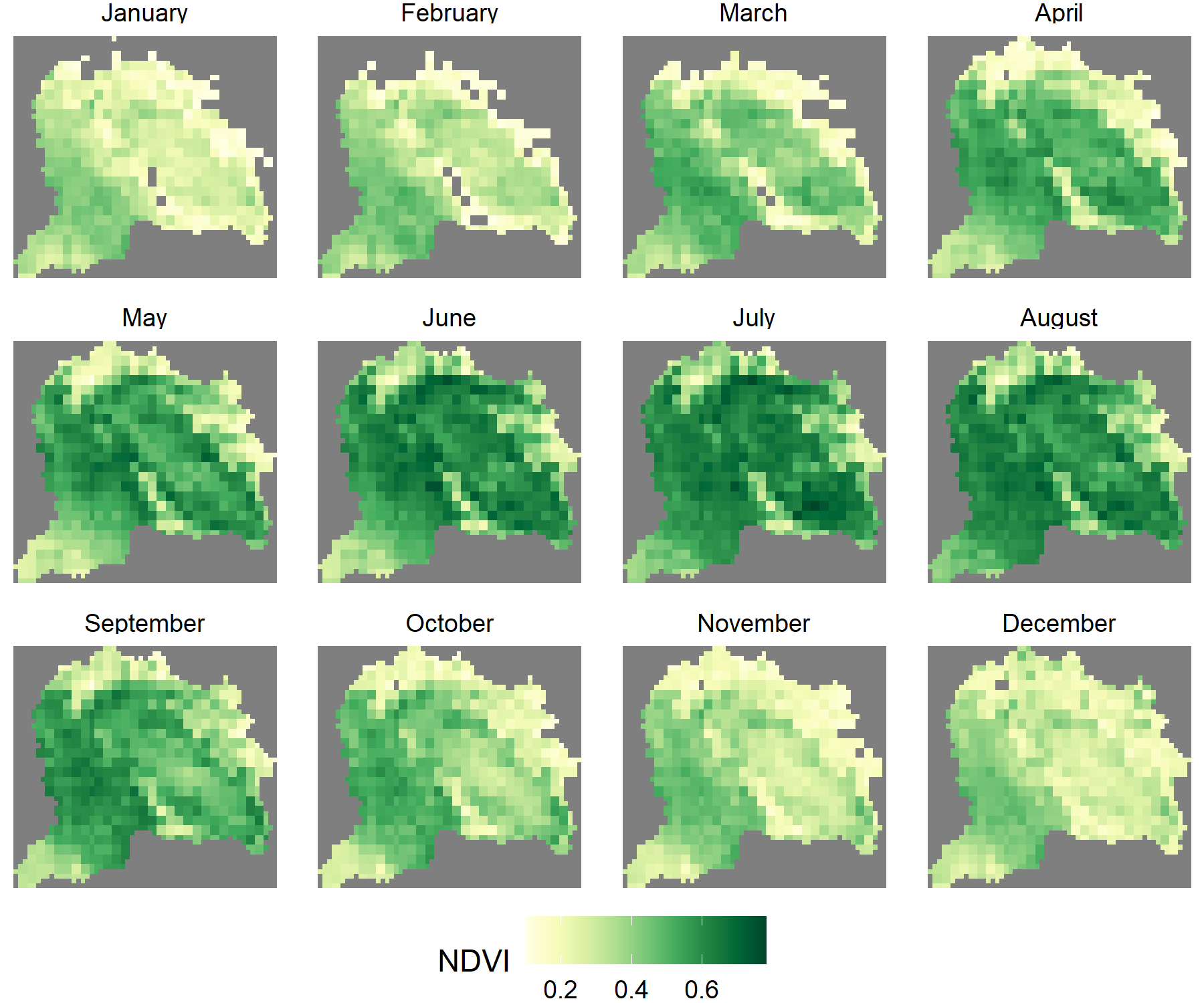

Supplement: S7 Fig — Average monthly NDVI over the UJRB during 1982 to 2015. (TIFF) [file pone.0271991.s007.tiff]

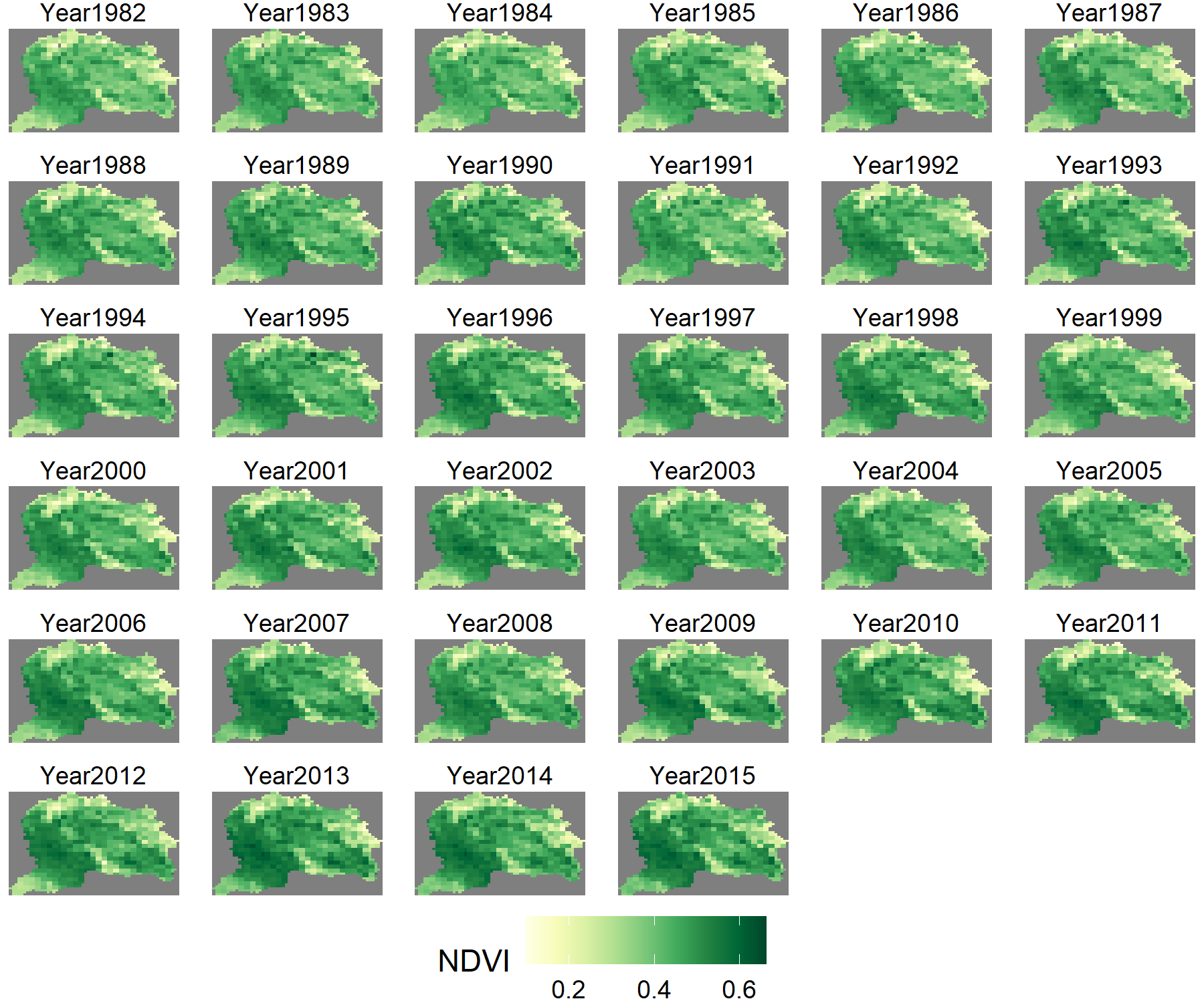

Supplement: S8 Fig — Average annual NDVI over the UJRB during 1982 to 2015. (TIFF) [file pone.0271991.s008.tiff]
